# Genetic risk factors for ischaemic stroke and its subtypes (the METASTROKE Collaboration): a meta-analysis of genome-wide association studies

**DOI:** 10.1016/S1474-4422(12)70234-X

**Published:** 2012-11

**Authors:** Matthew Traylor, Martin Farrall, Elizabeth G Holliday, Cathie Sudlow, Jemma C Hopewell, Yu-Ching Cheng, Myriam Fornage, M Arfan Ikram, Rainer Malik, Steve Bevan, Unnur Thorsteinsdottir, Mike A Nalls, WT Longstreth, Kerri L Wiggins, Sunaina Yadav, Eugenio A Parati, Anita L DeStefano, Bradford B Worrall, Steven J Kittner, Muhammad Saleem Khan, Alex P Reiner, Anna Helgadottir, Sefanja Achterberg, Israel Fernandez-Cadenas, Sherine Abboud, Reinhold Schmidt, Matthew Walters, Wei-Min Chen, E Bernd Ringelstein, Martin O'Donnell, Weang Kee Ho, Joanna Pera, Robin Lemmens, Bo Norrving, Peter Higgins, Marianne Benn, Michele Sale, Gregor Kuhlenbäumer, Alexander S F Doney, Astrid M Vicente, Hossein Delavaran, Ale Algra, Gail Davies, Sofia A Oliveira, Colin N A Palmer, Ian Deary, Helena Schmidt, Massimo Pandolfo, Joan Montaner, Cara Carty, Paul I W de Bakker, Konstantinos Kostulas, Jose M Ferro, Natalie R van Zuydam, Einar Valdimarsson, Børge G Nordestgaard, Arne Lindgren, Vincent Thijs, Agnieszka Slowik, Danish Saleheen, Guillaume Paré, Klaus Berger, Gudmar Thorleifsson, The Australian Stroke Genetics Collaborative, Wellcome Trust Case Control Consortium 2 (WTCCC2), Albert Hofman, Thomas H Mosley, Braxton D Mitchell, Karen Furie, Robert Clarke, Christopher Levi, Sudha Seshadri, Andreas Gschwendtner, Giorgio B Boncoraglio, Pankaj Sharma, Joshua C Bis, Solveig Gretarsdottir, Bruce M Psaty, Peter M Rothwell, Jonathan Rosand, James F Meschia, Kari Stefansson, Martin Dichgans, Hugh S Markus, on behalf of the International Stroke Genetics Consortium

**Affiliations:** a Stroke and Dementia Research Centre, St George's University of London, London, UK; b Wellcome Trust Centre for Human Genetics, University of Oxford, Oxford, UK; c Department of Cardiovascular Medicine, University of Oxford, Oxford, UK; d Centrw for Clinical Epidemiology and Biostatistics, School of Medicine and Public Health, University of Newcastle, and Center for Bioinformatics, Biomarker Discovery and Information-Based Medicine, Hunter Medical Research Institute, NSW, Australia; e Division of Clinical Neurosciences and Insititute of Genetics and Molecular Medicine, University of Edinburgh, Edinburgh, UK; f Clinical Trial Service Unit and Epidemiological Studies Unit, University of Oxford, Oxford, UK; g University of Maryland School of Medicine, Department of Medicine, Baltimore, MD, USA; h University of Texas Health Science Center at Houston, Houston, TX, USA; i Department of Epidemiology, Erasmus MC University Medical Center, Rotterdam, Netherlands; j Department of Neurology and Department of Radiology, Erasmus MC University Medical Center, Rotterdam, Netherlands; k Netherlands Consortium for Healthy Ageing, Leiden, Netherlands; l Institute for Stroke and Dementia Research, Klinikum der Universitát München, Ludwig-Maximilians-Universität, and Munich Cluster for Systems Neurology (SyNergy), Munich, Germany; m deCODE Genetics, Reykjavik, Iceland; n Faculty of Medicine, University of Iceland, Reykjavik, Iceland; o Laboratory of Neurogenetics, National Institute on Aging, Bethesda, MD, USA; p Department of Neurology, University of Washington, Seattle, WA, USA; q Department of Epidemiology, University of Washington, Seattle, WA, USA; r Cardiovascular Health Research Unit, Department of Medicine, University of Washington, Seattle, WA, USA; s Imperial College Cerebrovascular Research Unit (ICCRU), Imperial College London, London, UK; t Department of Cereberovascular Disease, Fondazione Istituto di Ricovero e Cura a Carattere Scientifico (IRCCS) Istituto Neurologico Carlo Besta, Milan, Italy; u Department of Biostatistics, Boston University School of Public Health, Boston, MA, USA; v Department of Neurology, University of Virginia, Charlottesville, VA, USA; w Department of Public Health Science, University of Virginia, Charlottesville, VA, USA; x Department of Neurology, Veterans Affairs Medical Center, Baltimore, MA, USA; y Department of Neurology, University of Maryland School of Medicine, MA, USA; z Division of Public Health Sciences, Fred Hutchinson Cancer Research Center, Seattle, WA, USA; aa Department of Neurology and Neurosurgery, Utrecht Stroke Center, Rudolf Magnus Institute of Neuroscience, University Medical Center Utrecht, Utrecht, Netherlands; ab Neurovascular Research Laboratory, Neurology and Medicine Departments, Universitat Autònoma de Barcelona and Institute of Research Vall d'Hebrón Hospital, Barcelona, Spain; ac Laboratory of Experimental Neurology, Brussels, Belgium; ad Department of Neurology, Division of Neurogeriatrics, Medical University Graz, Graz, Austria; ae Institute of Cardiovascular and Medical Sciences, University of Glasgow, Glasgow, UK; af Center for Public Health Genomics, University of Virginia, Charlottesville, VA, USA; ag Department of Neurology, University of Münster, Münster, Germany; ah National University of Ireland Galway, Galway, Ireland; ai Department of Public Health and Primary Care, University of Cambridge, Cambridge, UK; aj Department of Neurology, Jagiellonian University, Krakow, Poland; ak Laboratory of Neurobiology, Vesalius Research Center, VIB, Leuven, Belgium; al Experimental Neurology and Leuven Research Institute for Neurodegenerative Diseases (LIND), University of Leuven (KU Leuven), Leuven, Belgium; am Department of Neurology, University Hospital Leuven, Leuven, Belgium; an Department of Clinical Sciences Lund, Neurology, Lund University, and Department of Neurology, Skåne University Hospital, Lund, Sweden; ao Department of Clinical Biochemistry and The Copenhagen General Population Study, Herlev Hospital, Copenhagen University Hospital, and Faculty of Health Sciences, University of Copenhagen, Copenhagen, Denmark; ap Division of Cardiovascular Medicine, Department of Internal Medicine, University of Virginia, Charlottesville, VA, USA; aq Institute for Experimental Medicine, University of Kiel, Germany; ar Medical Research Institute, Ninewells Hospital and Medical School, University of Dundee, Dundee, UK; as Departamento Promoção da Saúde e Doenças Crónicas, Instituto Nacional de Saúde Dr Ricardo Jorge, Lisbon, Portugal; at Julius Center for Health Sciences and Primary Care, University Medical Center Utrecht, Utrecht, Netherlands; au Department of Psychology, and Centre for Cognitive Ageing and Cognitive Epidemiology, University of Edinburgh, Edinburgh, UK; av Instituto de Medicina Molecular, Faculdade de Medicina da Universidade de Lisboa, Lisbon, Portugal; aw Institute of Molecular Biology and Biochemistry, Medical University Graz, Graz, Austria; ax Department of Medical Genetics and Department of Epidemiology, University Medical Centre Utrecht, Utrecht, Netherlands; ay Program in Medical and Population Genetics, Broad Institute of Harvard and MIT, Cambridge, MA, USA; az Division of Genetics, Brigham and Women's Hospital, Harvard Medical School, Boston, MA, USA; ba Department of Neurology, Karolinska Institutet at Karolinska University Hospital, Huddinge, Sweden; bb Serviço de Neurologia, Centro de Estudos Egas Moniz, Hospital de Santa Maria, Lisbon, Portugal; bc Landspitali, University Hospital, Reykjavik, Iceland; bd The Copenhagen City Heart Study, Bispebjerg Hospital, Copenhagen University Hospital, Copenhagen, Denmark; be Centre for Non-Communicable Diseases, Karachi, Pakistan; bf Department of Medicine, University of Pennsylvania, PA, USA; bg Department of Pathology & Molecular Medicine and Department of Clinical Epidemiology & Biostatistics, McMaster University, Hamilton, ON, Canada; bh Institute of Epidemiology and Social Medicine, University of Münster, Münster, Germany; bi University of Mississippi Medical Center, Jackson, MS, USA; bj Department of Neurology, Massachusetts General Hospital, Boston, MA, USA; bk Centre for Translational Neuroscience and Mental Health Research, University of Newcastle, and Hunter Medical Research Institute, New Lambton, NSW, Australia; bl Department of Neurology, Boston University School of Medicine, Boston, MA, USA; bm Department of Epidemiology, Department of Medicine, and Department of Health Services, University of Washington, and Group Health Research Institute, Group Health Seattle, WA, USA; bn Stroke Prevention Research Unit, Nuffield Department of Clinical Neuroscience, University of Oxford, Oxford, UK; bo Center for Human Genetic Research, Massachusetts General Hospital, Boston, MA, USA; bp Department of Neurology, Mayo Clinic, Jacksonville, FL, USA

## Abstract

**Background:**

Various genome-wide association studies (GWAS) have been done in ischaemic stroke, identifying a few loci associated with the disease, but sample sizes have been 3500 cases or less. We established the METASTROKE collaboration with the aim of validating associations from previous GWAS and identifying novel genetic associations through meta-analysis of GWAS datasets for ischaemic stroke and its subtypes.

**Methods:**

We meta-analysed data from 15 ischaemic stroke cohorts with a total of 12 389 individuals with ischaemic stroke and 62 004 controls, all of European ancestry. For the associations reaching genome-wide significance in METASTROKE, we did a further analysis, conditioning on the lead single nucleotide polymorphism in every associated region. Replication of novel suggestive signals was done in 13 347 cases and 29 083 controls.

**Findings:**

We verified previous associations for cardioembolic stroke near *PITX2* (p=2·8×10^−16^) and *ZFHX3* (p=2·28×10^−8^), and for large-vessel stroke at a 9p21 locus (p=3·32×10^−5^) and *HDAC9* (p=2·03×10^−12^). Additionally, we verified that all associations were subtype specific. Conditional analysis in the three regions for which the associations reached genome-wide significance (*PITX2, ZFHX3*, and *HDAC9*) indicated that all the signal in each region could be attributed to one risk haplotype. We also identified 12 potentially novel loci at p<5×10^−6^. However, we were unable to replicate any of these novel associations in the replication cohort.

**Interpretation:**

Our results show that, although genetic variants can be detected in patients with ischaemic stroke when compared with controls, all associations we were able to confirm are specific to a stroke subtype. This finding has two implications. First, to maximise success of genetic studies in ischaemic stroke, detailed stroke subtyping is required. Second, different genetic pathophysiological mechanisms seem to be associated with different stroke subtypes.

**Funding:**

Wellcome Trust, UK Medical Research Council (MRC), Australian National and Medical Health Research Council, National Institutes of Health (NIH) including National Heart, Lung and Blood Institute (NHLBI), the National Institute on Aging (NIA), the National Human Genome Research Institute (NHGRI), and the National Institute of Neurological Disorders and Stroke (NINDS).

## Introduction

Stroke is one of the three most common causes of death, is a major cause of adult chronic disability,^1^ and represents an important cause of age-related cognitive decline and dementia. Conventional risk factors explain only a small proportion of all stroke risk.^2^ Evidence from studies of twins and family history suggests that genetic predisposition is important.^3^ In common with many other complex diseases, in which environmental risk factors are thought to interact with multiple genes, the identification of the underlying molecular mechanisms contributing to stroke risk has been a challenge. Candidate gene studies have produced few replicable associations.^4^ More recently, the genome-wide association study (GWAS) approach has transformed the genetics of other complex diseases and is just beginning to affect the study of stroke.^5^, ^6^

About 80% of stroke is ischaemic, whereas 20% is due to primary haemorrhage.^6^ Ischaemic stroke itself includes several subtypes with differing pathophysiological mechanisms, the most common of which are large-vessel disease stroke, small-vessel disease stroke, and cardioembolic stroke.^7^ Various genetic variants that predispose to risk factors for stroke have also been shown in GWAS to predispose to ischaemic stroke.^8^, ^9^, ^10^ Two loci associated with atrial fibrillation (*PITX2* and *ZFHX3*) were associated with cardioembolic stroke, whereas a locus on chromosome 9p21 originally associated with coronary artery disease was shown to be a risk factor for large-vessel stroke.^8^, ^9^, ^10^ The few novel stroke-associated loci reported to date have been mainly associated with stroke subtypes, rather than with the phenotype of ischaemic stroke. In Japanese populations, a variant in the protein kinase C family (*PRKCH*) was associated with small-vessel stroke.^11^ A meta-analysis of prospective population-based cohort studies reported an association with the 12p13 region, thought to be with the *NINJ2* gene, although this result was not replicated in a larger case-control sample.^12^, ^13^ Recently, the Wellcome Trust Case Control Consortium 2 (WTCCC2) GWAS in ischaemic stroke reported a novel association on chromosome 7p21 within the *HDAC9* gene, although it was associated only with large-vessel ischaemic stroke.^14^

GWAS in ischaemic stroke to date have used small discovery populations, with the largest including 3548 individuals.^14^ In other complex diseases, many additional associations have been detected as the discovery sample size has increased.^15^, ^16^, ^17^ This increase has usually been achieved by meta-analysis of independent datasets. Therefore, we established the METASTROKE collaboration to combine the available GWAS datasets of ischaemic stroke. Here, we describe the first paper from METASTROKE with a description of the constituent cohorts. Using this dataset, we attempted both to replicate previous GWAS associations with ischaemic stroke and to identify novel associations. Additionally, we determined whether stroke loci were specific to individual stroke subtypes.

## Methods

### Study design and participating studies

The discovery sample consisted of 15 cohorts of patients with ischaemic stroke who were of European ancestry from Europe, North America, and Australia, together with controls of matched ancestry. All studies used a case-control methodology. Most participating studies were cross-sectional, whereas four were in large, prospective, population-based cohorts (table 1).

**Table 1:** Description of cohorts used in analysis by study population

	**Number of cases**	**Number of CS cases**	**Number of LVD cases**	**Number of SVD cases**	**Number of controls**	**Ancestry**	**Study design**	**Genotyping**
**Discovery cohorts**								
ARIC	385	93	31	63	8803	European	Population-based	Affymetrix 6.0
ASGC	1162	240	421	310	1244	European	Cross-sectional	Illumina 610
BRAINS	361	29	120	97	444	European	Cross-sectional	Illumina 660
CHS	454	147	..	73	2817	European	Population-based	Illumina 370
deCODE	2391	399	255	240	26 970	European	Cross-sectional	Illumina 317/370
FHS	171	48	..	..	4164	European	Population-based	Affymetrix 550
GEOS	448	90	37	54	498	European	Cross-sectional	Illumina HumanOmni1
HPS	578	..	..	..	468	European	Cross-sectional	Illumina 610
HVH	566	88	61	173	1290	European	Cross-sectional	Illumina 370
ISGS/SWISS	1070	247	229	201	2329	European	Cross-sectional	Illumina 550/610/660
MGH-GASROS	516	169	95	38	1202	European	Cross-sectional	Affymetrix 6.0
Milano	372	25	74	65	407	European	Cross-sectional	Illumina 610/660
Rotterdam	367	..	..	..	5396	European	Population-based	Illumina 550
WTCCC2-Munich	1174	330	346	106	797	European	Cross-sectional	Illumina 660
WTCCC2-UK	2374	460	498	474	5175	European	Cross-sectional	Illumina 660
Total (discovery)	12 389	2365	2167	1894	62 004	..	..	..
**Replication cohorts**								
Barcelona	439	179	110	150	404	European	Cross-sectional	Sequenom
BSS	225	11	93	90	312	European	Cross-sectional	Sequenom
Copenhagen	730	..	..	..	1545	European	Cross-sectional	TaqMan
ESS	276	40	20	69	940	European	Cross-sectional	TaqMan/Illumina 610
Glasgow	675	125	91	150	940	European	Cross-sectional	Sequenom/Illumina 610
Go-Darts*	737	130	259	..	8424	European	Cross-sectional	Affymetrix 6.0/Illumina Cardio-metabochip
Graz	657	116	108	207	848	European	Cross-sectional	Sequenom/Illumina 610
Interstroke*	872	143	198	238	926	European	Cross-sectional	Illumina Cardio-metabochip
Krakow	1235	377	152	171	584	European	Cross-sectional	Sequenom
Leuven	458	195	83	63	391	European	Cross-sectional	Sequenom
Lund	424	140	21	94	466	European	Cross-sectional	Sequenom
Munster	1232	478	528	224	1053	European	Cross-sectional	Sequenom
Portugal	539	..	..	..	507	European	Cross-sectional	Sequenom
RACE (Pakistan)*	1322	225	195	189	1143	Pakistani	Cross-sectional	Illumina 660
SMART	623	30	368	195	6712	European	Population-based	Sequenom
Sweden	876	157	177	75	742	European	Cross-sectional	Sequenom
VISP*	1725	..	..	..	1047	European	Cross-sectional	Illumina HumanOmni1
WHI*	302	42	31	78	2099	European	Population-based	Illumina Omni-Quad
Total (replication)	13 347	2388	2434	1993	29 083	..	..	..

CS=cardioembolic stroke. LVD=large-vessel disease. SVD=small-vessel disease. ARIC=The Atherosclerosis Risk in Communities study. ASGC=Australian Stroke Genetics Collabarative. BRAINS=Bio-Repository of DNA in stroke. CHS=Cardiovascular Health Study. FHS=Framingham Heart Study. GEOS=Genetics of Early-Onset Stroke. HPS=Heart Protection Study. HVH=The Heart and Vascular Health Study. ISGS/SWISS=The Ischemic Stroke Genetics Study/Sibling with Ischaemic Stroke Study. MGH-GASROS=The MGH Genes Affecting Stroke Risk and Outcome Study. WTCCC2-Munich=The Wellcome Trust Case-Control Consortium II Munich. WTCCC2-UK=The Wellcome Trust Case-Control Consortium II UK. BSS=Belgium Stroke Study. ESS=Edinburgh Stroke Study. Go-Darts=Genetics of Diabetes Audit and Research in Tayside Study. RACE=Risk Assessment of Cerebrovascular Events Study, Pakistan. SMART=Second Manifestations of ARTerial disease. VISP=The Vitamin Intervention for Stroke Prevention Trial. WHI=The Women's Health Initiative.

* Contributed genome-wide data.

Additionally, 18 cohorts were analysed in the replication phase. These cohorts were included for replication only, most did not have GWAS data available; and those with GWAS data were not available at the time of the discovery analysis. 17 of the included cohorts contained individuals of solely European ancestry, and one contained individuals of Pakistani ancestry (table 1). Most cohorts (16) were cross-sectional, whereas two were population-based.

The appendix includes detailed descriptions of the design and clinical characteristics of the participating studies.

Stroke was defined as a typical clinical syndrome with radiological confirmation. Stroke subtyping was done with the Trial of Org 10172 in Acute Stroke Treatment (TOAST) classification system.^18^ Where subtyping was done, brain CT or MRI was undertaken for more than 95% of cases in all the discovery cohorts.

Participating studies were approved by relevant institutional review boards, and all participants gave written or oral consent for study participation, including genetic research, as approved by the local institutional body.

### Data imputation and statistical analysis

The 15 discovery cohorts used commercially available GWAS panels of single nucleotide polymorphisms (SNPs) from either Affymetrix (Santa Clara, CA, USA) or Illumina (San Diego, CA, USA). 14 of the 15 centres undertook genotype imputation with HapMap II,^19^ HapMap III,^20^ or 1000 Genomes^21^ as reference haplotype training sets. Every centre did genotypic quality control steps before imputation, including removal of ancestry outliers defined by principal component analysis and poorly typed individuals.

We used logistic regression for all cohorts with a cross-sectional study design to model the multiplicative SNP effects on risk for the dichotomous outcome of stroke against ancestry-matched controls, whereas we used Cox proportional-hazards models for the prospective studies to assess time to first stroke, fitting an additive model relating genotype dose to the stroke outcome. Where genotypes were imputed, SNPs were modelled as allele dosages. Of the discovery cohorts, four (of 15) centres used ancestry-informative principal components as covariates to correct for population stratification. All cohorts providing genome-wide data removed population outliers before imputation. After verifying strand alignment, filtering SNPs with minor allele frequency lower than 0·01, and removing poorly imputed SNPs across centres, we did a meta-analysis of the results of the association analyses from every centre using a fixed-effects inverse-variance weighted model using METAL.^22^

We sought further evidence for association with novel suggestively associated SNPs in new samples from 18 different cohorts. Of the 18 centres, six submitted in-silico genotype data and 12 undertook direct genotyping with the Sequenom (Sequenom, San Diego, CA, USA) or Taqman (Applied Biosystems, Foster City, CA, USA) platforms. All of the five replication cohorts contributing genome-wide data used principal components as covariates in their analyses. We did a meta-analysis of the results for the replication cohorts using a fixed-effects, inverse variance weighted method first for all datasets, and then for replication datasets of solely European ancestry. We determined whether SNPs were significantly associated in the replication population, and additionally, we combined results from the discovery and replication analyses using a fixed-effects, inverse-variance weighted approach.

We set the study-wide genome-wide significance level at p<5×10^−8^ to control the experiment-wide error rate to <5%. Following the example of previous GWAS studies,^15^ we set the level for suggestive significance at p<5×10^−6^.

First, we attempted to determine the evidence for association for the six loci reported previously from GWAS to be associated with ischaemic stroke (*HDAC9, PITX2, ZFHX3, NINJ2, PRKCH*, and 9p21).^8^, ^9^, ^10^, ^11^, ^12^, ^14^ After determining the evidence for association with the previously reported SNPs, we investigated whether any proxy SNPs were more significantly associated in the METASTROKE dataset. Because some loci had been identified in discovery populations included in METASTROKE, we initially did analyses for the whole dataset, and then we restricted analysis to the lead SNP for every locus in the METASTROKE cohorts that had not been included in the discovery phase of the initial publication. We set the significance level for independent replication at p<0·01, corresponding to Bonferroni corrected type 1 error <5% for the five SNPs (excluding *PRKCH*) tested.

As the SNP in *PRKCH* (rs2230500) underlying the previous association in Japanese cohorts^1^ is monomorphic in populations of European ancestry, we sought to identify any associations within this gene region, including the 50 kbp window upstream and downstream, in our large population of European ancestry. Using the modified Nyholt correction approach of Li and Ji on the 353 SNPs from the region, we estimated the effective number of SNPs tested to be 103·3.^23^ We therefore set the significance level at p<0·00048, corresponding to Bonferroni corrected type I error <5% for the effective SNPs tested.

We also did an analysis to determine whether the six previously reported variants were associated with stroke risk in prospective population-based studies. We did this analysis only for the known SNPs that had been analysed in a minimum of 100 cases in the prospective cohorts with incident stroke events for the relevant subtype.

For those associations we could confirm, we then did a conditional analysis within the associated region to identify any signal in the region that was independent of the lead SNP in every case. For every association, we selected regions used in the conditional analysis on the basis of adjacent recombination hotspots, meaning we analysed different numbers of SNPs for every locus (appendix). We used logistic regression in every centre, using imputed genotype dosages to model the effect of the lead SNP on risk as a covariate. We then did a meta-analysis of the results using a fixed-effects, inverse-variance weighted model. We used our suggestive significance threshold (p<5×10^−6^) to identify SNPs that were statistically independent of the lead SNP for every locus.

We then did a meta-analysis of the genome-wide study-specific analysed datasets to identify novel associations with ischaemic stroke and its subtypes. We did the primary association analyses for all ischaemic stroke and for the three major subtypes: cardioembolic stroke, large-vessel disease, and small-vessel disease. We did additional secondary analyses for young cases (younger than 70 years at first stroke) and for the phenotype of ischaemic stroke in each sex separately. We reused the same controls per centre for all analyses. Excluding the previously published associations, we considered all SNPs reaching suggestive significance (p<5×10^−6^) for replication. We examined SNPs for heterogeneity across datasets and attempted replication in independent datasets for the loci that were deemed plausible candidates for association with ischaemic stroke.

For a minor allele frequency of 0·25, we had 80% power to detect variants with a per-allele odds ratio (OR) greater than 1·11 for the all ischaemic stroke analysis, 1·23 for cardioembolic stroke, 1·24 for large-vessel disease, and 1·26 for small-vessel disease at p<5×10^−8^ in the discovery phase.

### Role of the funding source

The sponsors of the study had no role in study design, data collection, data analysis, data interpretation, or writing of the report. The corresponding author had full access to all the data in the study and had final responsibility for the decision to submit for publication.

## Results

**Figure 1:**
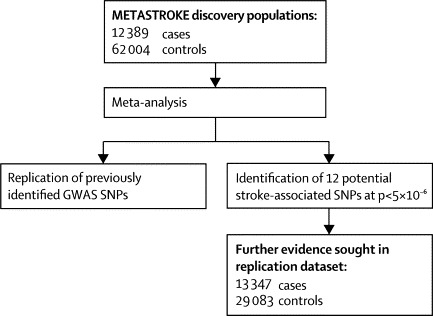
Flow diagram of METASTROKE analyses GWAS=genome-wide association study. SNP=single nucleotide polymorphism.

The discovery meta-analysis of ischaemic stroke phenotypes involved a total of 12 389 cases and 62 004 controls from 15 populations (table 1; figure 1).

The discovery meta-analysis confirmed associations at genome-wide significance levels for *HDAC9* with large-vessel disease, and for both *PITX2* and *ZFHX3* with cardioembolic stroke (table 2). For *PITX2, ZFHX3*, and *HDAC9* a proxy SNP was more significant in the METASTROKE dataset than the SNP from the original publication (original SNP shown in appendix). The 9p21 locus was associated with large-vessel disease with a similar OR (1·15, 95% CI 1·08–1·23, in METASTROKE) to that reported previously (1·21, 1·07–1·37),^10^ although it did not reach genome-wide significance (p=3·32×10^−5^). All four associations were subtype specific, being present only for a single stroke subtype (table 2). To determine the extent to which these results replicated the findings from the originally published associations, we repeated the meta-analysis, this time excluding the populations that contributed to the discovery phase of the original publication. For the *PITX2, ZFHX3, HDAC9*, and 9p21 loci, the associations were replicated in the independent METASTROKE samples (table 2). The population attributable risks in the METASTROKE discovery cohort were estimated as 5·8% for *PITX2* and 7·0% for *ZFHX3* in cardioembolic stroke, and 4·5% for *HDAC9* and 7·2% for 9p21 in large-vessel disease.

**Table 2:** METASTROKE association signals for SNPs identified in previous genome-wide association studies by gene and disease subtype

		**Chr**	**BP**	**SNP**	**RA**	**RAF**	**Full METASTROKE discovery sample**		Excluding cohorts used in previous discovery of relevant association*	
							OR (95% CI)	p value†	OR (95% CI)	p value†
*HDAC9*		7	19 015 913	rs2107595	A	0·16				
	IS	..	..	..	..	..	1·12 (1·07–1·17)	4·34×10^−6^	1·11 (1·05–1·17)	7·8×10^−5^
	LVD	..	..	..	..	..	1·39 (1·27–1·53)	2·03×10^−16^	1·39 (1·24–1·56)	3·15×10^−8^
	SVD	..	..	..	..	..	1·03 (0·93–1·14)	0·57	1·11 (0·96–1·29)	0·92
	CE	..	..	..	..	..	1·07 (0·98–1·17)	0·15	1·07 (0·96–1·19)	0·25
*PITX2*		4	111 937 516	rs6843082	G	0·21				
	IS	..	..	..	..	..	1·11 (1·06–1·15)	1·95×10^−7^	1·09 (1·04–1·14)	1·12×10^−4^
	LVD	..	..	..	..	..	1·06 (0·97–1·15)	0·17	1·03 (0·93–1·13)	0·61
	SVD	..	..	..	..	..	1·04 (0·96–1·14)	0·31	1·01 (0·90–1·13)	0·91
	CE	..	..	..	..	..	1·36 (1·27–1·47)	2·8×10^−16^	1·32 (1·23–1·44)	3·64×10^−12^
*ZFHX3*		16	71 626 169	rs879324	A	0·19				
	IS	..	..	..	..	..	1·05 (1·00–1·09)	0·037	1·06 (1·01–1·11)	0·021
	LVD	..	..	..	..	..	1·06 (0·98–1·16)	0·15	1·06 (0·96–1·17)	0·32
	SVD	..	..	..	..	..	0·99 (0·91–1·09)	0·94	1·01 (0·91–1·13)	0·81
	CE	..	..	..	..	..	1·25 (1·15–1·35)	2·28×10^−8^	1·25 (1·15–1·36)	1·53×10^−7^
*NINJ2*		12	645 460	rs11833579	A	0·22				
	IS	..	..	..	..	..	1·06 (1·02–1·10)	6·1×10^−4^	1·00 (0.96–1·05)	0·81
	LVD	..	..	..	..	..	0·99 (0·91–1·08)	0·87	0·99 (0·91–1·08)	0·79
	SVD	..	..	..	..	..	0·98 (0·90–1·08)	0·79	0·99 (0·90–1·08)	0·79
	CE	..	..	..	..	..	1·04 (0·97–1·13)	0·27	1·00 (0·92–1·09)	0·95
9p21		9	22 105 959	rs2383207	G	0·52				
	IS	..	..	..	..	..	1·04 (0·76–1·41)	0·024	1·03 (0·99–1·07)	0·16
	LVD	..	..	..	..	..	1·15 (1·08–1·23)	3·32×10^−5^	1·15 (1·04–1·27)	5·69×10^−3^
	SVD	..	..	..	..	..	1·02 (0·96–1·10)	0·48	1·03 (0·93–1·14)	0·61
	CE	..	..	..	..	..	0·96 (0·91–1·03)	0·24	1·02 (0·92–1·14)	0·61
*PRKCH*										
	IS	14	61 077 900	rs2246700	A	0·84	1·07 (1·02–1·12)	0·0049	..	..
	LVD	14	60 894 555	rs12587610	G	0·31	1·11 (1·03–1·21)	0·0046	..	..
	SVD	14	61 114 037	rs2255146	G	0·82	1·22 (1·03–1·43)	0·0175	..	..
	CE	14	60 988 886	rs3825655	C	0·95	1·31 (1·00–1·71)	0·0475	..	..

Chr=chromosome. BP=base position. SNP=single nucleotide polymorphism. RA=risk allele. RAF=risk allele frequency. OR=odds ratio. IS=all ischaemic strokes. LVD=large vessel disease. SVD=small vessel disease. CE=cardioembolic stroke.

* Statistics shown are after removal of discovery populations showing an association between the gene and stroke from original publications—ie, deCODE excluded for *PITX2, ZFHX3*;^8^, ^9^ WTCCC2-UK and WTCCC-Munich excluded for *HDAC9*;^14^ WTCCC2-UK and WTCCC2-Munich, ISGS/SWISS, GEOS, and MGH-GASROS excluded for *CDKN2a/CDKN2b* (9p21);^10^ Rotterdam, ARIC, FHS, and CHS excluded for *NINJ2*.^12^

† One-sided p value.

The *NINJ2* locus showed nominal evidence of association with all ischaemic stroke when all populations were included (table 2). However, no evidence was noted for association with the *NINJ2* locus when the original discovery populations were excluded (table 2).

To estimate the effect of these associations in prospective population-based studies, we had a sufficient number of stroke cases for the analysis in only the cardioembolic subtype (n=376). We noted ORs similar to those identified in the overall case-control study for both *PITX2* (1·26, 95% CI 1·05–1·52, in prospective studies and 1·36, 1·27–1·47, in case-control analysis) and *ZFHX3* (1·23, 0·98–1·55, in prospective studies and 1·25, 1·15–1·35, in case-control analysis), although this similarity was significant only for *PITX2* (appendix).

We found no significant associations between the *PRKCH* gene region and all ischaemic strokes or with the three main subtype analyses. Table 2 provides details of the most strongly associated SNPs in every subtype for this locus.

**Figure 2:**
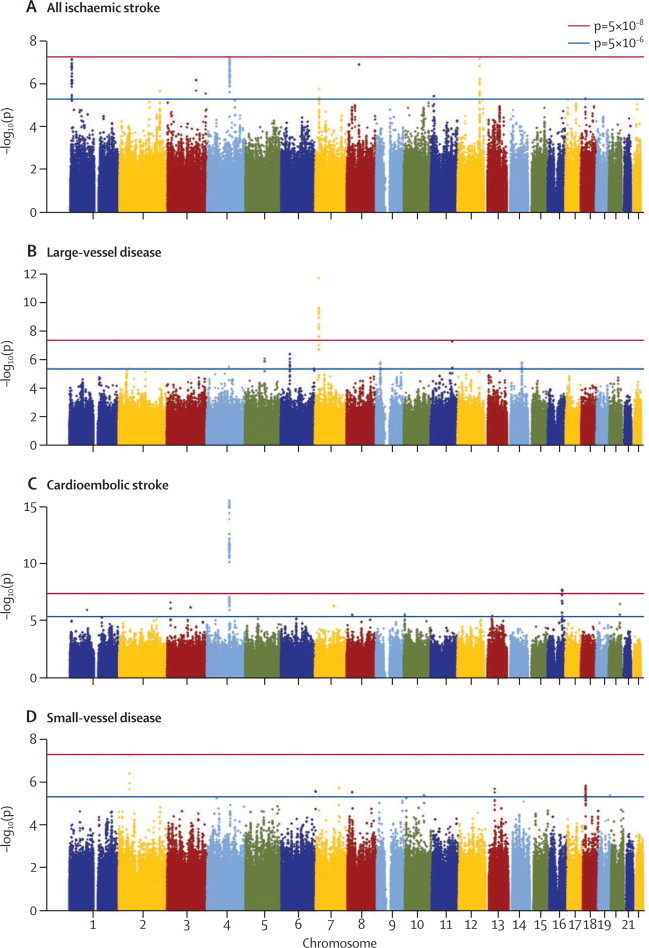
Manhattan plots of –log_10_(p) against genomic position for principal analyses (A) All ischaemic stroke. (B) Large-vessel disease. (C) Cardioembolic stroke. (D) Small-vessel disease. Genome-wide meta-analysis association results by genomic position for the four main analyses.

For those loci for which we confirmed genome-wide significance (*PITX2, ZFHX3*, and *HDAC9*), we did conditional analyses. After conditioning on the lead SNP in the given region, no SNP showed significance at p<0·01 in *PITX2* or *ZFHX3*, and no SNP showed significance at p<0·005 in *HDAC9*. Furthermore, all other SNPs in the regions that were associated at p<5×10^−8^ in the main analysis showed no significance (p>0·05) in any of the analyses after conditioning on the lead SNP. Figure 2 shows plots of –log_10_(p values) against genomic position in the selected regions for the unconditional and conditional analyses.

**Figure 3:**
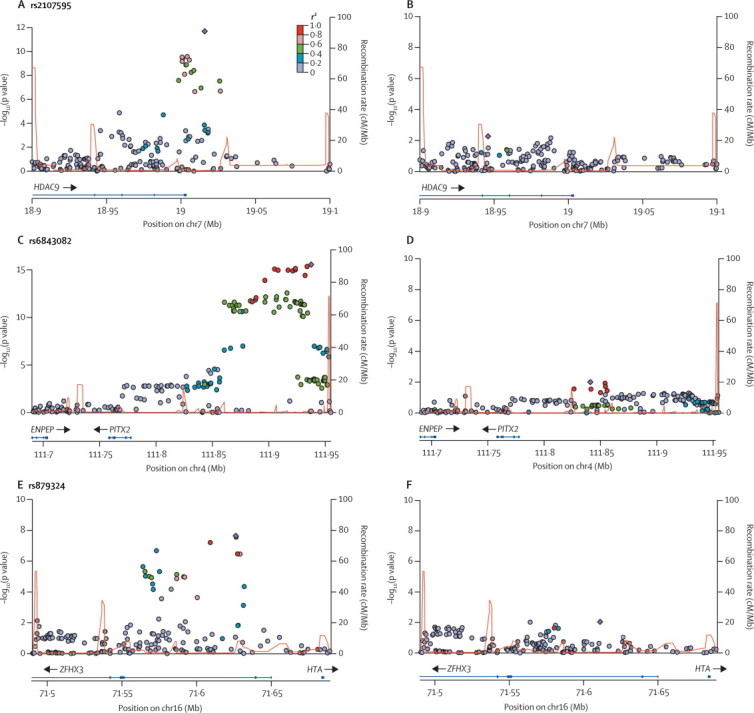
Plots of conditional analysis regions before and after conditioning on lead SNP SNP=single nucleotide polymorphism. Plots of association signals around loci investigated in conditional analyses in subtypes in which they were discovered for the meta-analysed discovery samples. SNPs are coloured on the basis of their correlation (r^2^) with the labelled top SNP, which has the smallest p value in the region. The fine-scale recombination rates estimated from HapMap data are marked in red, with genes marked below by horizontal blue lines. Arrows on the horizontal blue lines show the direction of transcription, and rectangles are exons. (A,C,E) Regions from discovery meta-analyses. (B,D,F) Same regions as A,C,E after conditioning on the lead SNP from the region.

We selected a total of 12 novel SNPs for testing in the independent replication cohort: three associated with all ischaemic stroke, five associated with specific stroke subtypes, and two each associated with young stroke and female stroke. Four of these SNPs showed associations close to genome-wide significance in the discovery cohort: rs225132 in the *ERRF11* gene and rs17696736 in the *NAA25* (C12orf30) gene with all ischaemic stroke (p=6·3×10^−8^ and 5·9×10^−8^, respectively), rs7937106 in *ALKBH8* with large-vessel disease (p=5·9×10^−8^), and rs13407662 on chromosome 2p16.2 (p=5·2×10^−8^) in an intergenic region with small-vessel disease. The remaining SNPs were identified at the suggestive significance level of p<5×10^−6^. Table 3 shows details of these SNPs, including stroke subtypes with which they were associated, and significance levels. These 12 novel SNPs were taken forward for replication in an additional 13 347 cases and 29 083 controls. Figure 3 shows the plots of –log_10_(p values) by chromosomal location for the analysis of all stroke and the three main subtypes.

**Table 3:** Association signals for SNPs selecting for testing in the independent replication cohort by subtype

	**Chr**	**SNP**	**Candidate gene**	**RA**	**RAF**	**p**_discovery_**; OR**_discovery_**(95% CI)**	**All replication samples**		**Replication in European descent individuals only**	
							p_replication_; OR_replication_ (95% CI)	p_combined_	p_replication_; OR_replication_ (95% CI)	p_combined_
IS	1	rs225132	*ERRFI1*	T	0·82	6·27×10^−8^; 1·12 (1·07–1·17)	0·16; 0·97 (0·92–1·01)	1·65×10^−3^	0·11; 0·96 (0·92–1·01)	1·91×10^−3^
IS	12	rs17696736	*NAA25 (C12orf30)*	G	0·42	5·97×10^−8^; 1·10 (1·06–1·14)	0·59; 1·01 (0·97–1·05)	1·92×10^−5^	0·60; 1·01 (0·97–1·05)	1·69×10^−5^
IS	3	rs16851055	*SPSB4*	G	0·81	6·34×10^−7^; 1·12 (1·07–1·17)	0·20; 1·03 (0·98–1·08)	6·23×10^−6^	0·25; 1·03 (0·98–1·08)	7·76×10^−6^
CS	3	rs6763538	*OXNAD1*	T	0·04	2·89×10^−7^; 1·47 (1·27–1·69)	0·69; 1·04 (0·87–1·24)	2·68×10^−5^	0·59; 1·05 (0·88–1·25)	1·36×10^−5^
LVD	11	rs7937106	*ALKBH8*	C	0·16	5·85×10^−8^; 1·68 (1·40–2·03)	0·66; 1·04 (0·87–1·25)	3·93×10^−5^	0·65; 1·05 (0·85–1·31)	1·42×10^−4^
LVD	6	rs556621	*..*	T	0·33	4·63×10^−7^; 1·20 (1·12–1·28)	0·46; 1·03 (0·96–1·10)	5·33×10^−5^	0·37; 1·03 (0·96–1·11)	2·43×10^−5^
SVD	18	rs7407640	*AFG3L2*	A	0·21	2·20×10^−6^; 1·23 (1·13–1·34)	0·99; 1·00 (0·91–1·10)	4·54×10^−4^	0·57; 0·97 (0·88–1·07)	1·16×10^−3^
SVD	2	rs13407662	*..*	T	0·04	5·18×10^−8^; 1·95 (1·53–2·48)	0·28; 1·16 (0·89–1·51)	1·97×10^−6^	0·36; 1·14 (0·86–1·53)	1·88×10^−6^
FS	3	rs7432308	*..*	T	0·15	1·63×10^−6;^ 1·16 (1·09–1·24)	0·15; 0·95 (0·88–1·51)	4·80×10^−3^	0·37; 0·96 (0·89–1·05)	9·13×10^−4^
FS	12	rs2238151	*ALDH2*	T	0·66	1·03×10^−6^; 1·13 (1·08–1·19)	0·26; 1·03 (0·98–1·09)	8·62×10^−6^	0·22; 1·04 (0·98–1·11)	3·98×10^−6^
YS	7	rs12703165	*PRKAG2*	G	0·82	5·63×10^−7^; 1·20 (1·12–1·29)	0·49; 0·98 (0·93–1·04)	0·012	0·89; 1·00 (0·94–1·06)	1·81×10^−3^
YS	8	rs4875812	*ARHGEF10*	G	0·55	1·40×10^−6^; 1·16 (1·10–1·23)	0·87; 1·00 (0·97–1·03)	0·034	0·94; 1·00 (0·97–1·03)	0·024

Chr=chromosome. SNP=single nucleotide polymorphism. RA=risk allele. RAF=risk allele frequency. p_discovery_=one-sided p value in discovery cohorts. OR_discovery_=odds ratio in discovery cohorts. p_replication_,=one-sided p value in replication cohorts. OR_replication_=odds ratio in replication cohorts. p_combined_=one-sided p value in all cohorts combined. IS=all ischaemic stroke. CS=cardioembolic stroke. LVD=large-vessel disease. SVD=small-vessel disease. FS=female-only stroke. YS=young stroke.

None of the novel SNPs reached genome-wide significance on combination of the discovery and replication data. This result was the same when replication analysis was restricted to individuals of European ancestry (table 3). There was significant heterogeneity (p<0·05) for all of the SNPs in the combined analysis. We had sufficient sample size to obtain 80% power to confirm each of the 12 loci (appendix).

## Discussion

METASTROKE is the first large meta-analysis of stroke GWAS data (panel). The METASTROKE collaboration brings together GWAS data from more than 12 000 cases of ischaemic stroke and 60 000 controls from 15 cohorts all of European ancestry. In this first analysis from the dataset, we confirmed four of five previously described associations with ischaemic stroke in populations of European ancestry, including replication in an independent non-overlapping sample of the dataset not included in the original GWAS. All these associations were with specific subtypes of ischaemic stroke, emphasising the genetic heterogeneity of the disease. Additionally, we identified several promising novel associations, some of which were close to genome-wide significance in the discovery cohorts, but these were not confirmed in our replication population.

Panel **Research in context**

**Systematic review**

As part of the International Stroke Genetics Consortium we had access to several genome-wide association datasets for ischaemic stroke, including both published and unpublished studies. To identify other studies, we searched PubMed on July 30, 2012, for published genome-wide association studies in ischaemic stroke with the terms “ischaemic stroke” and “genome wide association”. The search returned studies already included by the consortium members. No further studies with ischaemic stroke as a primary endpoint were identified.

**Interpretation**

This is the largest analysis of genetic data for ischaemic stroke. This study provides evidence that common genetic variation has a role in the pathogenesis of ischaemic stroke. The genetic associations identified so far are with specific stroke subtypes, suggesting that the different subtypes of ischaemic stroke have different risk factor profiles and pathophysiological mechanisms, with potential implications for all areas of stroke research.

Our results provide further robust data supporting an association between two gene regions (*PITX2* and *ZFHX3*) and cardioembolic stroke, and a further two (*HDAC9* and 9p21) with large-vessel stroke although the 9p21 locus did not reach genome-wide significance. In all cases, these associations were present in the dataset as a whole, and also when those samples used in the original discovery cohorts that identified associations with ischaemic stroke were excluded.

Both *PITX2* and *ZFHX3* were originally identified as risk factors for atrial fibrillation.^8^, ^9^ Atrial fibrillation is a major risk factor for stroke, particularly in the elderly, and therefore their association with ischaemic stroke is not unexpected. Our results confirm this association and clearly show that it is limited to the cardioembolic stroke subtype. Furthermore, we were able to show an association between *PITX2* and ischaemic stroke in prospective cohorts. A potential bias is that a variant that is in fact associated with mortality rate after acute stroke and not with stroke risk might seem to be related to risk; for cross-sectional studies in a disease such as stroke, which has substantial early mortality, death might occur before or soon after hospital admission before samples are taken. In a prospective study, such cases are included as the sample was taken at recruitment to the study and therefore before the onset of stroke.

By contrast, the *HDAC9* and 9p21 associations were specific to large-vessel stroke, and not present with other stroke subtypes. An association with the 9p21 locus was first associated with myocardial infarction and coronary artery disease but has now been associated more widely with other arterial diseases such as aneurysms and ischaemic stroke.^10^, ^24^ *HDAC9* was recently identified in the WTCCC2 ischaemic stroke study as a novel association with ischaemic stroke,^14^ having not previously been shown in GWAS analyses of ischaemic heart disease.

For the *PITX2, ZFHX3*, and *HDAC9* associations, we did a conditional analysis to establish whether the lead SNP that we had identified was sufficient to model all of the associations within that region, or whether other independent genetic variants were associated with disease. In every case, no significant association remained after controlling for the lead SNP, suggesting that all the signal in each region can be attributed to one risk haplotype.

A meta-analysis of prospective cohort studies reported an association between ischaemic stroke and a SNP in the 12p13 region, although this was not replicated in an independent study.^13^ The underlying gene was suggested to be *NINJ2*.^12^ This association was present in the METASTROKE discovery cohort, but this cohort contained the datasets in which the original association had been determined. When these datasets were excluded, there was no evidence of any associations.

In a Japanese population, a variant in *PRKCH* has been associated with small-artery disease, a stroke subtype that is particularly common in this ethnic group.^11^ This association was confirmed in a prospective study with relatively few stroke endpoints, and also in a Chinese population.^25^, ^26^ Interestingly, an association was also suggested with cerebral haemorrhage, which shares some underlying pathological similarities with cerebral small-vessel disease causing lacunar infarction. The association has not yet been examined in other ancestral groups. The SNP is monomorphic in European populations and therefore we were unable to examine whether the association was present in our population. However, we assessed all SNPs at this chromosomal region and noted no evidence of any association in our population of European ancestry.

We identified associations at four loci that were near genome-wide significance in the discovery cohort and had not been associated with stroke in previous studies: SNPs in the *ERRF11* and *NAA25* (C12orf30) genes with all ischaemic stroke, a SNP in *ALKBH8* with large-vessel stroke, and rs13407662 on chromosome 2p16.2 in an intergenic region with small-vessel disease. We took these four forward, with an additional eight of the strongest associations that had not reached genome-wide significance, to replication in an independent sample. None of the associations replicated. Our replication sample contained a cohort of patients of Pakistani ancestry, but, restriction of our analysis to individuals of European ancestry did not alter the results.

The same risk allele of SNP rs17696736 in the *NAA25* gene has previously been associated with type 1 diabetes in a large genome-wide association study.^27^ Other SNPs in this 12q24 region have also been implicated in several of related phenotypes including microcirculation in vivo, platelet count, and blood pressure.^28^, ^29^, ^30^ None of the other three associations near to genome-wide significance have previously been associated with cardiovascular or neurological disease.

Our inability to replicate any of the novel associations we identified in the discovery phase could be explained by various factors. All non-imputed SNPs in all cohorts were checked for Hardy-Weinberg equilibrium and standard quality control measures were done, including checking for sex mismatch on the basis of three genotypic markers, but we cannot rule out confounding by other means. For example, many of the 12 replication cohorts only directly genotyped the 12 replication SNPs. First, this type of analysis provides no means of adjustment for ancestry-informative principal components, which could lead to results being adversely affected by population structure. Second, our strategy of attempting replication with one SNP from each region might not have been optimum. In regions such as the 12q24 locus, where the linkage disequilibrium patterns are complex, attempting replication in multiple SNPs might have proved more fruitful. Furthermore, one SNP (rs13407662) associated with small-vessel disease in the discovery phase failed genotyping in more than half of the replication cohorts. Genotyping multiple SNPs at this locus might have avoided this issue. We also cannot rule out confounding because of other environmental factors or phenotypic heterogeneity. Although phenotyping was done using the TOAST classification system, interpretation of exact classification criteria and definitions can differ across countries and studies, which becomes more of an issue when there are many smaller cohorts, such as in the replication phase of this study. Varying cohort study designs might also increase heterogeneity in large-scale meta-analyses.

Our results show that although genetic variants can be detected with ischaemic stroke, all associations we were able to confirm were specific to a stroke subtype. This finding has two implications. First, to maximise success of genetic studies in ischaemic stroke, detailed stroke subtyping is needed. Second, it implies that different pathophysiological mechanisms are associated with different stroke subtypes and, therefore, drug treatments might have different effects in different stroke subtypes. Most trials of secondary prevention in stroke have included all strokes, with limited stroke subtyping, and further studies with the detailed subtyping would be required to show different pharmacological profiles.

METASTROKE brings together GWAS data from most groups working in the area of stroke genetics worldwide. This paper describes the details of every population and represents the first analysis of the datasets. Various additional GWAS studies in stroke are currently taking place or have recently been completed, including a recently published GWAS in an Australian population, which confirmed an association at a 6p21.1 locus with large-artery atherosclerotic stroke.^31^ The addition of these data might lead to identification of further novel associations with ischaemic stroke.
