# Corrections

**DOI:** 10.1016/S1474-4422(15)00155-6

**Published:** 2015-08

*Traylor M, Farrall M, Holliday EG, et al, on behalf of the International Stroke Genetics Consortium. Genetic risk factors for ischaemic stroke and its subtypes (the METASTROKE Collaboration): a meta-analysis of genome-wide association studies.* Lancet Neurol *2012; **11:** 951–62*—In this Article, the last sentence of the VISP section in the acknowledgments section should have been “Control data for comparison with VISP cases were obtained through the database of genotypes and phenotypes (dbGAP) High Density SNP Association Analysis of Melanoma: Case-Control and Outcomes Investigation (phs000187.v1.p1). Research support to collect data and develop an application to support this project was provided by 3P50CA093459, 5P50CA097007, 5R01ES011740, and 5R01CA133996.” This correction has been made to the online version as of July 13, 2015.

