# Supplementary material for: Genetic risk factors for ischaemic stroke and its subtypes (the METASTROKE Collaboration): a meta-analysis of genome-wide association studies
Source: Lancet Neurol. 2012 Nov;11(11):951–62. doi: 10.1016/S1474-4422(12)70234-X (PMC3490334; doi:10.1016/S1474-4422(12)70234-X)
Supplement: Supplementary appendix [file mmc1.pdf]

THE LANCET Neurology

Supplementary webappendix

This webappendix formed part of the original submission and has been peer reviewed. We post it as supplied by the authors.

Supplement to: Traylor M, Farrall M, Holliday EG, et al, on behalf of the International Stroke Genetics Consortium. Genetic risk factors for ischaemic stroke and its subtypes (the METASTROKE Collaboration): a meta-analysis of genome-wide association studies. *Lancet Neurol* 2012; published online Oct 5. http://dx.doi.org/10.1016/S1474-4422(12)70234-X.

Genetic risk factors for ischaemic stroke and its subtypes (the METASTROKE Collaboration): a meta-analysis of genome-wide association studies

Web Extra Material

Clinical phenotyping and stroke subtyping

In two discovery centers (Rotterdam, HPS) and three replication centers (Copenhagen, VISP, Portugal) data on subtypes was not available and these cohorts were only included in analyses of all ischaemic stroke and sex specific analyses.

Statistical Analysis across studies

Analysis was restricted to SNPs that met the QC criteria in at least six centers for the young stroke analysis as well as the LVD, CE, and SVD subtypes. All centres provided data for the all ischaemic stroke and sex specific analyses, so SNPs present in at least eight centers were considered for analysis. The number of SNPs remaining after QC in each analysis was approximately 2·4M, roughly corresponding to the SNPs from the HapMap II SNP set. Genomic Inflation factor correction was used per center to correct for over-dispersion. After meta-analysis, statistical heterogeneity was evaluated using Cochran's test (Q-test). Manhattan and QQ-plots were generated using the R statistical software package (http://www.R-project.org). Plots of the $-\log_{10}$(p-values) by genomic position for associations of statistical significance were generated using LocusZoom (http://csg.sph.umich.edu/locuszoom/).

Discovery analysis

Four of the discovery cohorts used ancestry-informative prinicipal components as covariates to correct for population stratification (ISGS/SWISS, GEOS, ASGC, BRAINS). Age and sex were included as covariates in two centres (ISGS/SWISS and BRAINS), sex was used as a covariate in one centre (MGH-GASROS) and one center used recruitment phase (1 or 2) as a covariate (GEOS). In all other centres no covariates were included.

Replication analysis

For all centers, SNPs were removed that failed testing for Hardy-Weinberg equilibrium at $p<1x10^{-4}$. The cases from Edinburgh and Glasgow were from cohorts approximately 60 miles apart geographically. We therefore combined the cases and analysed against a common control set from the same geographical region (Lothian Birth Cohort 1936). Age and sex were included as covariates in four centres (RACE, WHI, Interstroke, GO-Darts) and ancestry-informative principal components were included in five centres (RACE, WHI, Interstroke, GO-Darts, VISP).

Population Attributable Risks

Population attributable risks were estimated using the following formula:

$$PAR\% = \frac{100\,x\,p(OR-1)}{p(OR-1)+1}$$

where OR is the odds ratio and p is the prevalence of the risk allele.

Supplementary Table 1: Description of conditional analysis regions used in analysis of established loci

Gene	Lead SNP	Chromosome	SNP Base Position	Region from (recombination rate peak*)	Region to (recombination rate peak)
HDAC9	rs2107595	7	19,015,913	18,900,000 (0·3 cM)	19,100,000 (0·7 cM)
ZFHX3	rs879324	16	71,626,169	71,490,000 (0·5 cM)	71,690,000 (0·3 cM)
PITX2	rs6843082	4	111,937,516	111,690,000 (0·6 cM)	111,955,000 (0·4 cM)

*approximate recombination rate, as measured in centimorgans (cM) for HapMap II CEU population.

Supplementary Table 2: Description of SNP associations in METASTROKE for replicated SNPs from original publication (where different to METASTROKE top SNP)

Gene	CHR	BP	SNP	RA	RAF	Subtype	OR (95% CI)	P
HDAC9	7	18,998,460	rs11984041	A	0·10	LVD	1·35 (1·21–1·50)	$2{\cdot}6 \times 10^{-8}$
PITX2	4	111,929,618	rs2200733	A	0·13	CE	1·34 (1·23–1·46)	7.0×10^{-11}
ZFHX3	16	71,609,121	rs2106261	A	0·19	CE	1·23 (1·14–1·33)	6.2×10^{-8}

RA, risk allele; RAF, risk allele frequency; CE, cardioembolic stroke; LVD, large vessel disease; OR, odds ratio

Supplementary Table 3: Description of associations with established cardioembolic stroke SNPs in prospective population based cohort studies

Gene	No· cases	No· controls	SNP	RA	RAF	Subtype	OR (95% CI)	P
PITX2	378	16745	rs6843082	G	0·22	CE	1·26 (1·05–1·52)	0·013
ZFHX3	378	16745	rs879324	A	0·17	CE	1·23 (0·98–1·55)	0·079

RA, risk allele; RAF, risk allele frequency; CE, cardioembolic stroke; OR, odds ratio

Supplementary Table 4: Power Calculations of sample size required to achieve 80% power to replicate SNPs from discovery phase in Replication cohorts

Subtype	SNP	RAF	OR in discovery	Sample size required for 80% power to replicate*	No. cases in replication cohort
IS	rs225132	0.82	1.12	6054	13347
IS	rs17696736	0.42	1.10	4647	13347
IS	rs16851055	0.81	1.12	5794	13347
CE	rs6763538	0.04	1.47	1448	2388
LVD	rs7937106	0.16	1.68	232	2434
LVD	rs556621	0.33	1.20	1360	2434
SVD	rs7407640	0.21	1.23	1359	1993
SVD	rs13407662	0.04	1.95	412	1993
FS	rs7432308	0.15	1.16	3427	5948
FS	rs2238151	0.66	1.13	3100	5948
YS	rs12703165	0.82	1.20	2293	7407
YS	rs4875812	0.15	1.16	1879	7407

*calculations based on OR and RAF from discovery phase, assuming at least twice as many controls than cases. P-value for replication set at $p<0.0042$ corresponding to Bonferonni corrected type 1 error <5% for the 12 SNPs tested.

LVD, large vessel disease; CE, cardioembolic stroke; IS, all ischaemic stroke; SVD, small vessel disease; FS, female stroke; YS, young stroke; RAF, risk allele frequency; OR, odds ratio;

Supplementary Figure 1 - QQ-plots for main analyses after removal of known loci

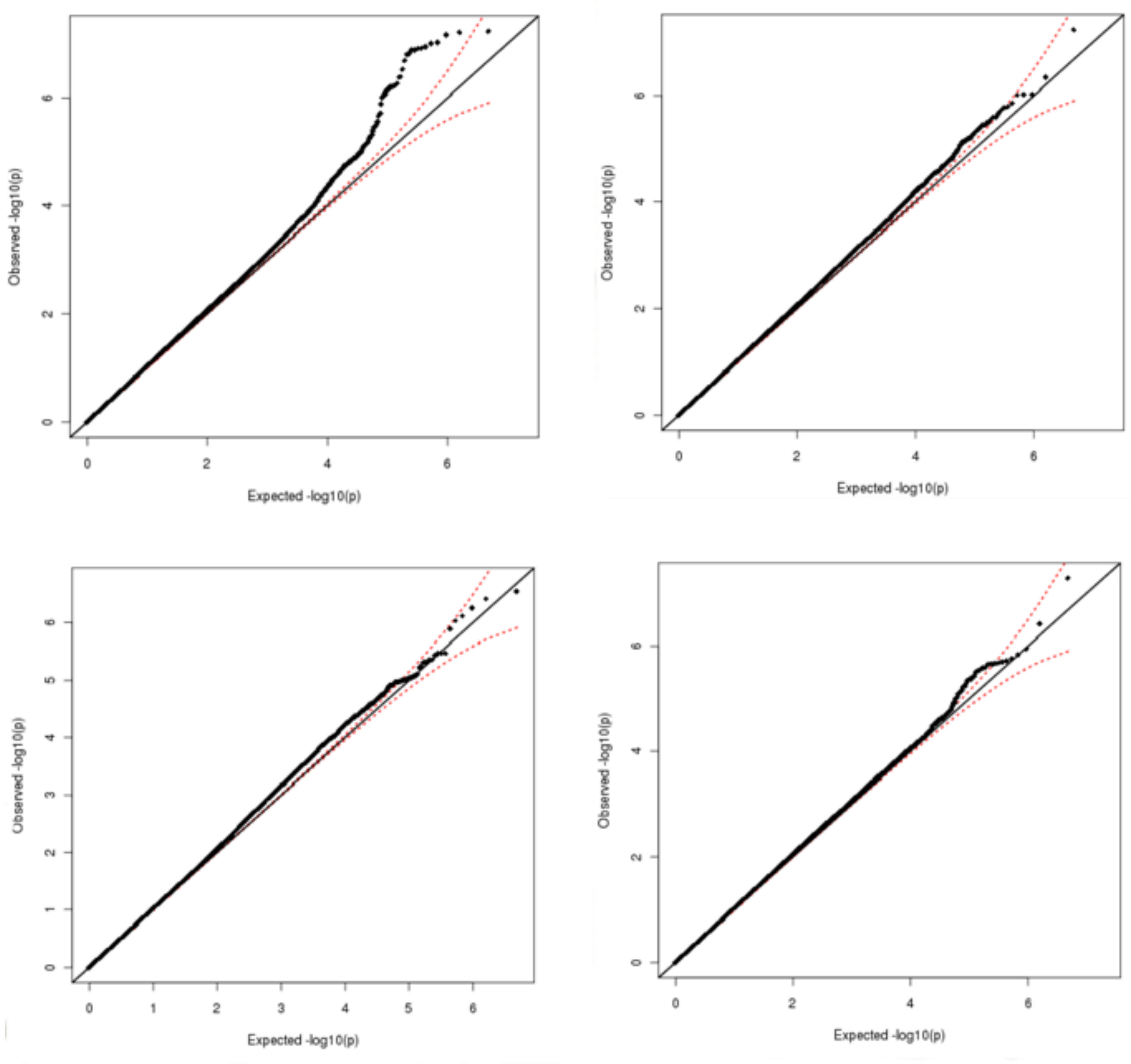

(a)-(d) clockwise from top left: (a) QQ-plot of all SNPs in all ischaemic stroke analysis after removal of SNPs from 9p21, HDAC9, ZFHX3, and PITX2 loci (inflation factor=1.040); (b) QQ-plot of remaining SNPs from large vessel disease phenotype after removal of SNPs from HDAC9 and 9p21 loci (inflation factor=1.036); (c) QQ-plot of all SNPs from small vessel disease phenotype (inflation factor=1.024); (d) QQ-plot of remaining SNPs from cardioembolic stroke phenotype after removal of SNPs from PITX2 and ZFHX3 loci (inflation factor=1.027).

Supplementary Figures 2-13 - Forest Plots of 12 SNPs selected for replication in the 18 replication populations

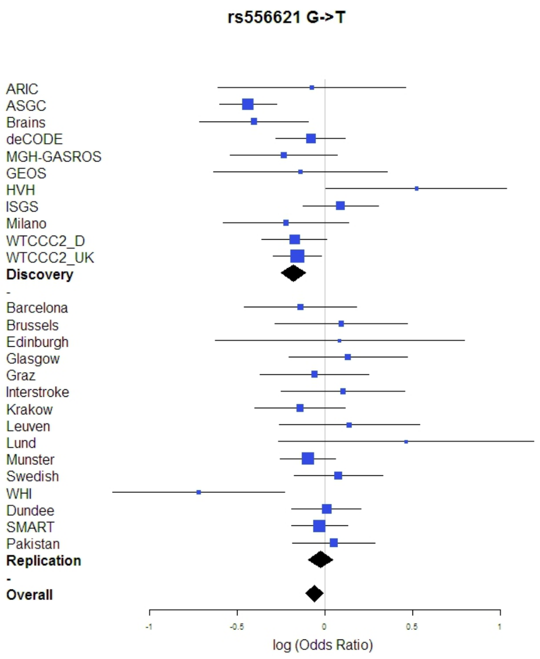

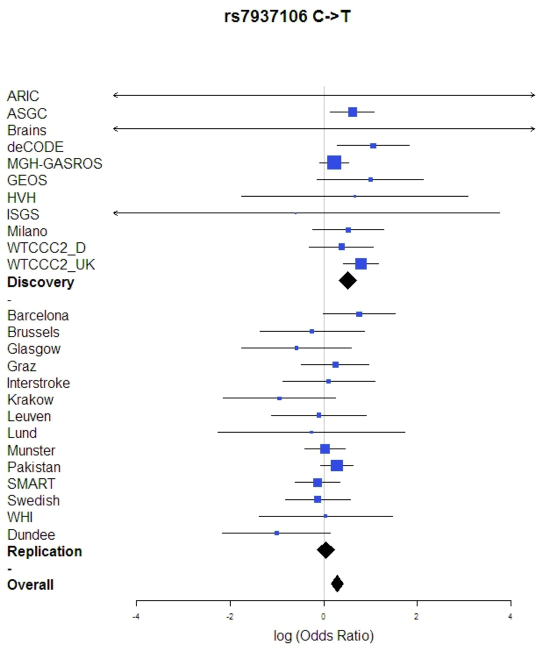

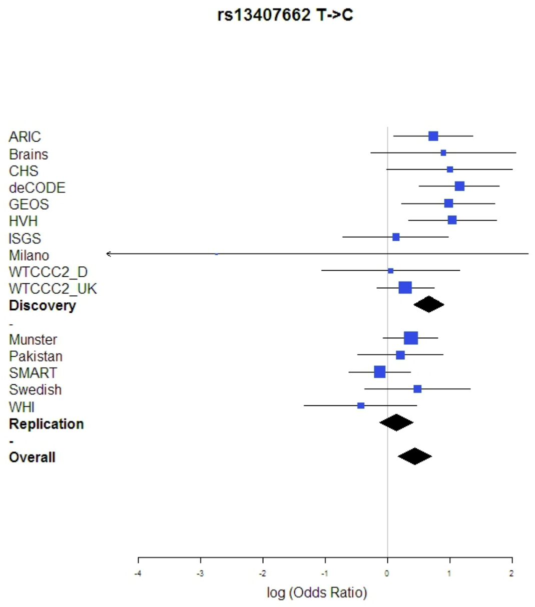

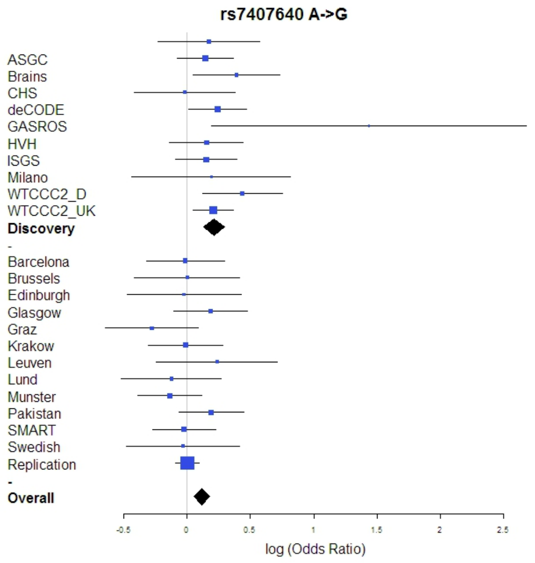

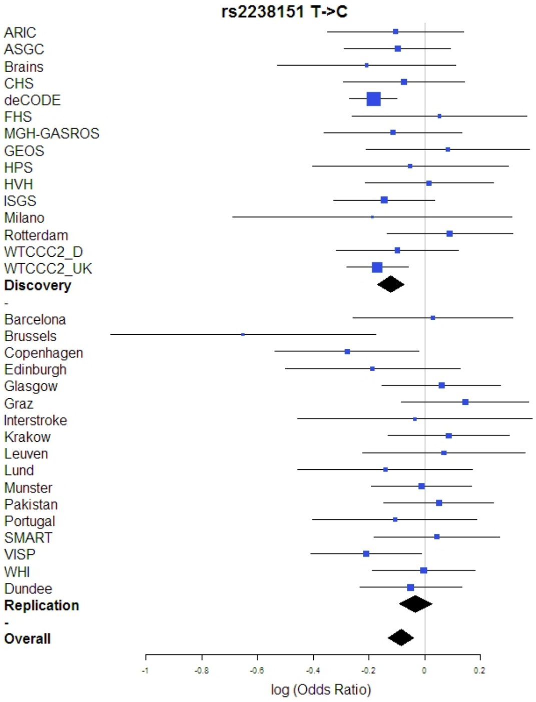
rs2238151 T->C
ARIC
ASGC
Brains
CHS
deCODE
FHS
MGH-GASROS
GEOS
HPS
HVH
ISGS
Milano
Rotterdam
WTCCC2_D
WTCCC2_UK
Discovery
-
Barcelona
Brussels
Copenhagen
Edinburgh
Glasgow
Graz
Interstroke
Krakow
Leuven
Lund
Munster
Pakistan
Portugal
SMART
VISP
WHI
Dundee
Replication
-
Overall
-1
-0.8
-0.6
-0.4
-0.2
0
0.2
log (Odds Ratio)

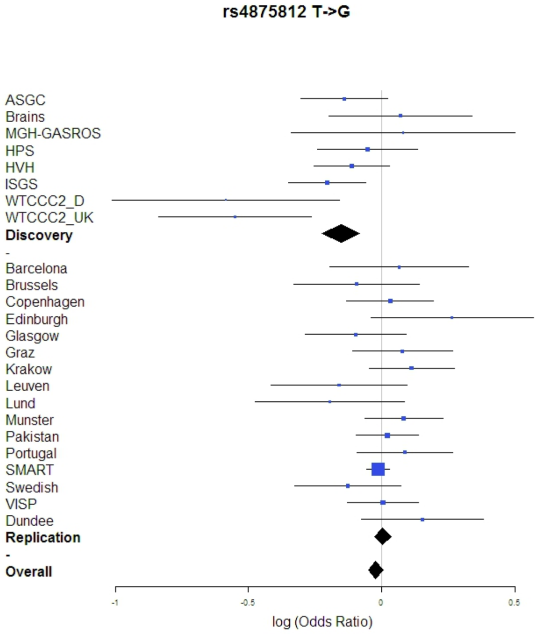
rs4875812 T->G
ASGC
Brains
MGH-GASROS
HPS
HVH
ISGS
WTCCC2_D
WTCCC2_UK
Discovery
-
Barcelona
Brussels
Copenhagen
Edinburgh
Glasgow
Graz
Krakow
Leuven
Lund
Munster
Pakistan
Portugal
SMART
Swedish
VISP
Dundee
Replication
-
Overall
-1
-0.5
0
0.5
log (Odds Ratio)

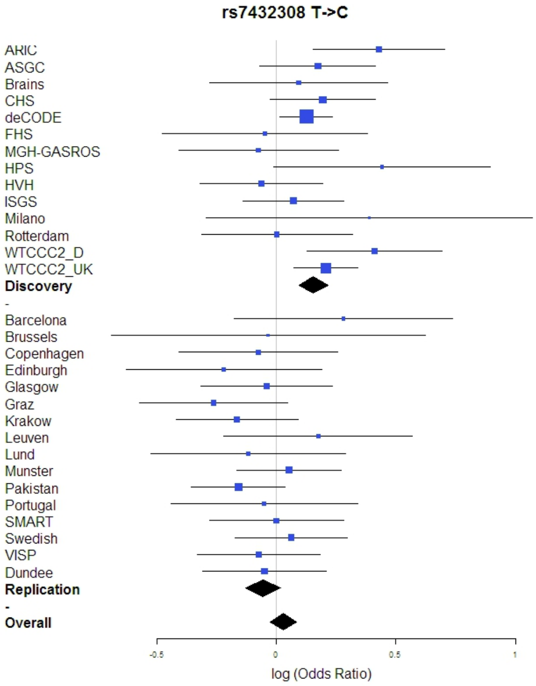
rs7432308 T->C
ARIC
ASGC
Brains
CHS
deCODE
FHS
MGH-GASROS
HPS
HVH
ISGS
Milano
Rotterdam
WTCCC2_D
WTCCC2_UK
Discovery
-
Barcelona
Brussels
Copenhagen
Edinburgh
Glasgow
Graz
Krakow
Leuven
Lund
Munster
Pakistan
Portugal
SMART
Swedish
VISP
Dundee
Replication
-
Overall
-0.5
0
0.5
1
log (Odds Ratio)

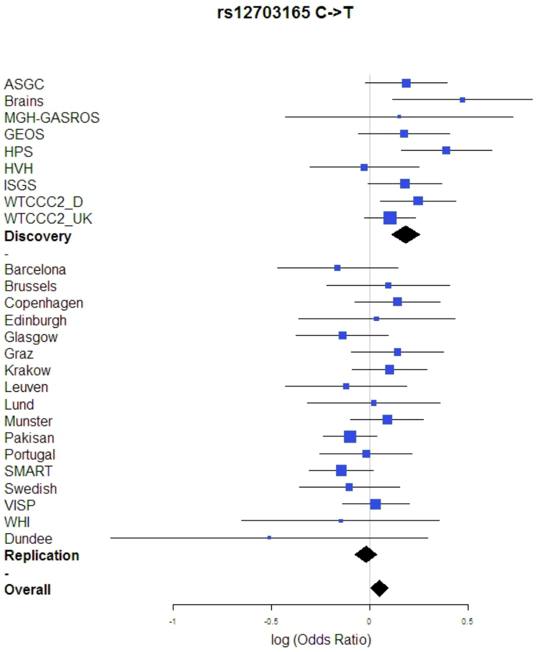
rs12703165 C->T
ASGC
Brains
MGH-GASROS
GEOS
HPS
HVH
ISGS
WTCCC2_D
WTCCC2_UK
Discovery
-
Barcelona
Brussels
Copenhagen
Edinburgh
Glasgow
Graz
Krakow
Leuven
Lund
Munster
Pakisan
Portugal
SMART
Swedish
VISP
WHI
Dundee
Replication
-
Overall
-1
-0.5
0
0.5
log (Odds Ratio)

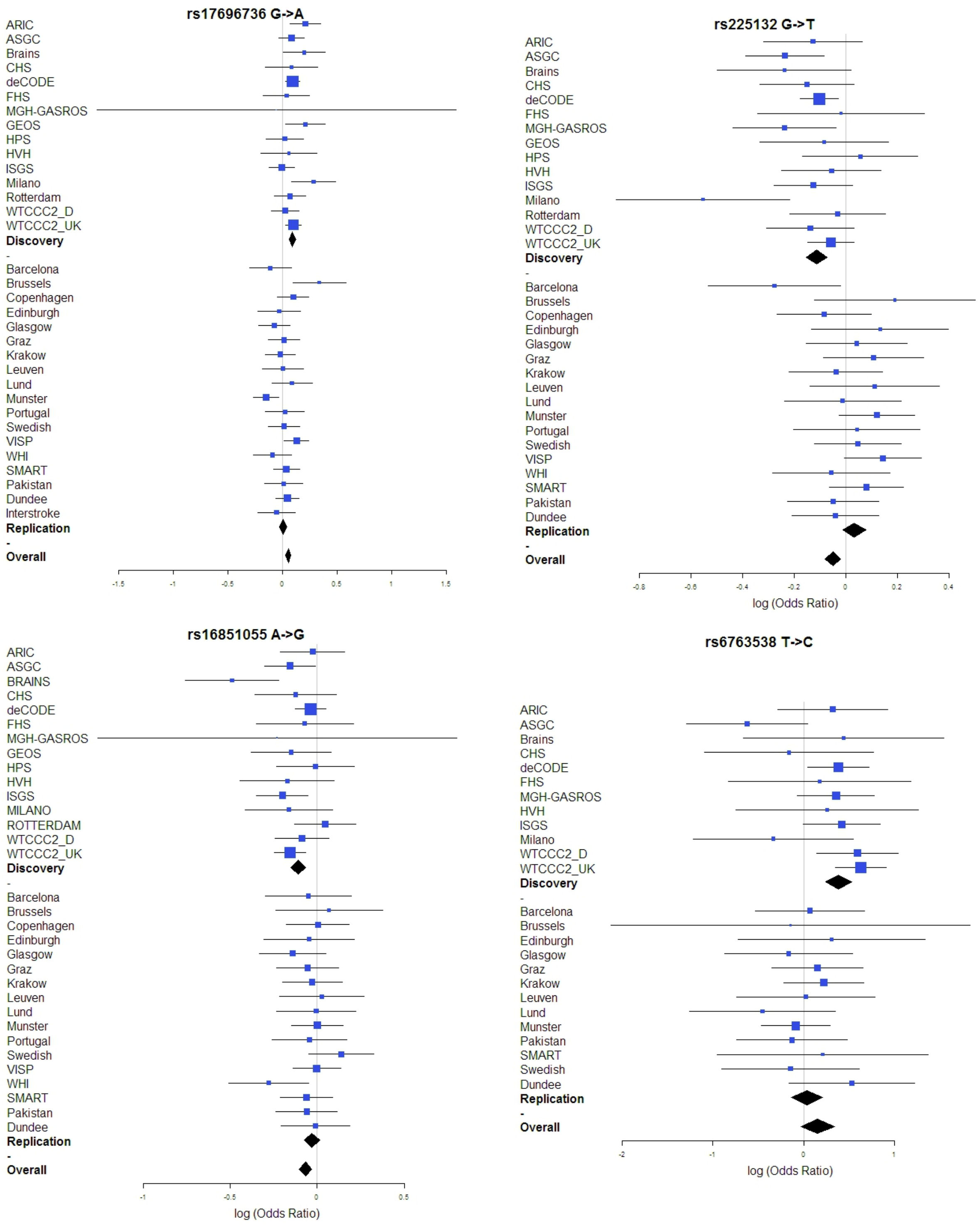

Note: Dundee known otherwise as GO-Darts.

Supplementary Table 4: Proportion of stroke cases with investigations performed in the discovery cohorts.

Cohort	Number of Cases	Brain imaging with CT or MRI (%)	MRI (%)	ECG (%)	Echocardiographic Imaging (%)	Extracranial cerebral artery imaging (%)
ARIC	385	98	-	-	-	-
ASGC	1162	97.4	43.0	59.6	20.0	5.0
BRAINS*	387	100	30.8	35.4	46.5	90.4
CHS	454	94.5	31.3	-	-	-
deCODE	2391	100	-	100	-	100
FHS	171	96.5**	75.4	100	70.2	84.8
GEOS	448	100	89.5	86.4	77.7	90.4
HPS	578	-	-	-	-	-
HVH	566	96.5	32.7	-	44.9	70.1
ISGS / SWISS	1070	100	83	89	76	85
Milano	372	100	86.7	100	20.0	100
MGH-GASROS*	533	100	60	47	54	100
Rotterdam	367	100	-	100	-	-
WTCCC2-Edinburgh (UK) *	713	100	25.9	100	36.9	93.8
WTCCC2- London (UK) *	1288	100	50.5	100	43.1	98.0
WTCCC2- Oxford (UK) *	884	100	26.9	100	26.4	100
WTCCC2- Munich*	1228	100	83	100	80	100

ARIC=The Atherosclerosis Risk in Communities study. ASGC=Australian Stroke Genetics Collabarative. BRAINS=Bio-Repository of DNA in stroke. CHS=Cardiovascular Health Study. FHS=Framingham Heart Study. GEOS=Genetics of Early-Onset Stroke. HPS=Heart Protection Study. HVH=The Heart and Vascular Health Study. ISGS/SWISS=The Ischemic Stroke Genetics Study/Sibling with Ischaemic Stroke Study. MGH-GASROS=The MGH Genes Affecting Stroke Risk and Outcome Study. WTCCC2=The Wellcome Trust Case-Control Consortium II. CT=Computed Tomography. MRI=Magnetic Resonance Imaging. ECG=Electrocardiogram. "-"=data not available, not recorded or performed only as clinically indicated. Extracranial cerebral arterial imaging includes carotid and vertebral artery ultrasound, or computed tomographic angiography, or magnetic resonance angiography. * values given are for individuals prior to genotypic QC steps. **Persons without imaging had autopsy-verified stroke.

Supplementary Table 5: Discovery Cohort Characteristics

Sample	Sample size					Age (mean ± SD)					Male Gender (%)					CT/MRI Brain Imaging (%)
	IS	SVD	CE	LVD	controls	IS	SVD	CE	LVD	controls	IS	SVD	CE	LVD	controls	
ARIC	385	63	93	31	8803	57.3 ± 5.3	55.3 ± 6.2	57.4 ± 5.0	57.8 ± 4.3	54.1 ± 5.7	60.3	58.7	62.4	58.1	46.5	98
ASGC	1162	310	240	421	1244	72.9 ± 13.2	77.5 ± 13.1	81.2 ± 12.1	73.2 ± 12.3	70.2 ± 12.1	59.2	57.4	50.4	68.7	50.2	100
BRAINS	361	97	29	120	444	74.4 ± 14.2	73.9 ±15.4	74.8 ±11.7	77.7 ± 11.9	65+[a]	55.9	52.5	51.6	52.5	35.8	100
CHS	454	73	147	-	2817	81.6 ± 6.11	74.3 ± 6.1	73.6 ± 5.7	-	85.8 ± 5.64	45.0	34.2	42.9	-	45.0	94.5
deCODE	2391	240	399	255	26970	73.3 ± 12.1	68.8 ± 10.2	71.2 ± 10.9	70.5 ± 9.3	57.3 ± 21.4	55.0	56.5	60.4	61.2	38.0	100
FHS	171	-	48	-	4164	79.6 ±10.5	-	83.3 ± 9.1	-	71.9± 11.9	43.3	-	39.6	-	45.1	96.5
GEOS	448	54	90	37	498	41.0 ± 7.0	44.3 ± 4.1	39.2 ± 7.5	45.2 ± 2.7	39.5 ± 6.7	61.4	72.2	58.9	67.6	56.6	100
HPS	578	-	-	-	468	64.9 ± 7.5	-	-	-	59.2 ± 9.3	75.0	-	-	-	67.0	-
HVH	566	173	88	61	1290	69.2 ± 8.6	67.6 ± 9.3	71.1 ± 7.7	67.9 ± 9.4	66.6 ± 9.1	33.7	32.9	33.0	44.3	47.7	97.2
ISGS/ SWISS	1070	201	247	229	2329	66.5 ± 13.6	64.6 ± 13.6	67.8 ± 14.4	69.1 ± 12.2	64.8 ± 12.6	57.2	60.3	53.7	59.2	48.0	100
MGH-GASROS	516	38	169	95	1202	66.7 ± 14.5	65.7 ± 14.2	66.2 ± 13.2	66.8 ± 13.8	47.5 ± 8.5	60.3	63.2	68.0	58.9	59.1	100
Milano	372	65	25	74	407	57.4 ± 15.6	56.2 ± 17.3	59.9 ± 15.0	63.5 ± 10.9	50.8 ± 8.1	62.8	56.7	68.0	76.0	87.7	100
Rotterdam	367	-	-	-	5396	70.8 ± 7.5	-	-	-	69.0 ± 9.0	45.2	-	-	-	40.3	100
WTCCC2-Munich	1174	106	330	346	797	66.7 ± 12.9	65.1±12.9	71.7± 12.1	65.7 ± 10.8	62.7 ± 10.9	61.9	72.6	52.1	70.7	51.4	100
WTCCC2-UK	2374	474	460	498	5175	72.2 ± 12.5	75.4 ± 12.5	69.6 ± 11.7	68.2 ± 10.8	~52[b]	53.8	52.3	62.1	66.2	49.5	100

ARIC=The Atherosclerosis Risk in Communities study. ASGC=Australian Stroke Genetics Collabarative. BRAINS=Bio-Repository of DNA in stroke. CHS=Cardiovascular Health Study. FHS=Framingham Heart Study. GEOS=Genetics of Early-Onset Stroke. HPS=Heart Protection Study. HVH=The Heart and Vascular Health Study. ISGS/SWISS=The Ischemic Stroke Genetics Study/Sibling with Ischaemic Stroke Study. MGH-GASROS=The MGH Genes Affecting Stroke Risk and Outcome Study.

WTCCC2=The Wellcome Trust Case-Control Consortium II. IS=Ischaemic Stroke. SVD=small vessel disease. LVD=large vessel disease. CE=cardioembolic stroke; "-"=information not available or not relevant. [a] All controls were aged 65 years or older at the time of genotyping. No further information was available. [b] The approximate age at genotyping of the 2738 controls from the 1958 Birth Cohort. Age was not available for the remaining controls.

Ascertainment and clinical assessment of participants in discovery studies

Participating studies were approved by relevant institutional review boards, and all participants gave informed consent for study participation, including genetic research.

The Atherosclerosis Risk in Communities Study (ARIC)
The ARIC study is a prospective population-based study of atherosclerosis and clinical atherosclerotic diseases in 15,792 men and women, including 11,478 non-Hispanic white participants, drawn from 4 U.S. communities (Suburban Minneapolis, Minnesota; Washington County, Maryland; Forsyth County, North Carolina, and Jackson, Mississippi). In the first three communities, the sample reflects the demographic composition of the community. In Jackson, only black residents were enrolled. Ancestry was self-reported during an interview. Over 99% self-identified as either white or black. Only self-identified whites were included in the analyses.[1]

Hospitalized strokes that occurred by December 31, 2007 (median follow-up, 18.7 years) were included in the analyses. During annual telephone contacts, trained interviewers asked each ARIC participant to list all hospitalizations during the past year. Hospital records for any hospitalizations indentified were then obtained. Moreover, all local hospitals annually provided lists of stroke discharges (International Classification of Diseases, Ninth Revision, Clinical Modification codes 430 to 438), which were surveyed for ARIC participant discharges. Details on quality assurance for ascertainment and classification of stroke are described elsewhere.[2] http://hyper.ahajournals.org/content/57/2/167.full - ref-10 Briefly, the stroke diagnosis was assigned according to criteria adapted from the National Survey of Stroke.[3] Strokes secondary to trauma, neoplasm, hematologic abnormality, infection, or vasculitis were excluded, and a focal deficit lasting <24 hours was not considered to be a stroke. Out-of-hospital stroke was not ascertained and validated; thus, these potential stroke events are not included. Strokes were classified into hemorrhagic stroke (subarachnoid and intracerebral hemorrhage) and ischemic stroke (thrombotic and embolic brain infarction). A stroke was classified as ischemic when a brain CT or MRI revealed acute infarction and showed no evidence of hemorrhage. All definite ischemic strokes were further classified as lacunar, nonlacunar thrombotic, or cardioembolic on the basis of the recorded neuroimaging results. A stroke was classified as “lacunar” when 2 criteria were met: (1) typical location of the infarct (basal ganglia, brain stem, thalamus, internal capsule, or cerebral white matter); and (2) infarct size of ≤2 cm or unstated size.[4] Definite or probable “cardioembolic” stroke required either (1) autopsy evidence of an infarcted area in the brain and a source of possible cerebral emboli in a vessel or the presence of an embolus in the brain or (2) medical record evidence of a possible noncarotid source of embolus such as moderate or greater valvular heart disease, atrial fibrillation, cardiac or arterial procedure (eg, cardiac catheterization, open heart surgery, cerebral angiography, and carotid endarterectomy), or intracardiac thrombus. Definite or probable ischemic strokes that were not classified as lacunar or cardioembolic, including atherothrombotic and unclassified thrombotic strokes, were labeled “nonlacunar.” Hemorrhagic strokes identified by ARIC were censored at the time of their occurrence.

Australian Stroke Genetics Collaborative (ASGC)
ASGC stroke cases comprised European-ancestry stroke patients admitted to four clinical centres across Australia (The Neurosciences Department at Gosford Hospital, Gosford, New South Wales (NSW); the Neurology Department at John Hunter Hospital, Newcastle, NSW; The Queen Elizabeth Hospital, Adelaide ; and the Royal Perth Hospital, Perth) between 2003 and 2008. Stroke was defined by WHO criteria as a sudden focal neurologic deficit of vascular origin, lasting more than 24 hours and confirmed by imaging such as computerised tomography (CT) and/or magnetic resonance imaging (MRI) brain scan. Other investigative tests such as electrocardiogram, carotid doppler and trans-oesophageal echocardiogram were conducted to define IS mechanism as clinically appropriate. Cases were excluded from participation if aged <18 years, diagnosed with haemorrhagic stroke or transient ischemic attack rather than IS, or were unable to undergo baseline brain imaging. Based on these criteria, a total of 1230 IS cases were included in the current study. IS subtypes were assigned using TOAST criteria, based on clinical, imaging and risk factor data.

ASGC controls were participants in the Hunter Community Study (HCS), a population-based cohort of individuals aged 55-85 years, predominantly of European Caucasian ancestry and residing in the

Hunter Region, NSW, Australia. Detailed recruitment methods for the HCS have been previously described [31]. Briefly, participants were randomly selected from the NSW State electoral roll and contacted by mail between 2004 and 2007. Consenting participants completed five detailed self-report questionnaires and attended the HCS data collection centre, at which time a series of clinical measures were obtained. A total of 1280 HCS participants were genotyped for the current study.

All study participants gave informed consent for participation in genetic studies. Approval for the individual studies was obtained from relevant institutional ethics committees.

Bio-Repository of DNA in Stroke (BRAINS)

The Bio-Repository of DNA in Stroke (BRAINS) is an international study recruiting highly phenotyped patients with stroke. For the purposes of the current work all patients were Caucasians. Diagnosis of stroke was confirmed using positive imaging (MRI or CT) and ischemic stroke subtypes were assigned using TOAST criteria, based on clinical, imaging and risk factor data. Controls were European-Ancestry, stroke-free participants from the shared WTCCC controls, a prospectively collected cohort of individuals born in 1958 (1958 Birth Cohort). The cohort has been described in detail elsewhere.[5]

Cardiovascular Health Study (CHS)

The CHS is a population-based cohort study of risk factors for coronary heart disease (CHD) and stroke in adults ≥65 years conducted across four field centers in the United States.[6] The original predominantly white cohort of 5,201 persons (4,964 whites) was recruited in 1989-1990 from a random sample of people on Medicare eligibility lists and an additional 687 African-Americans were enrolled subsequently (1992-93) for a total sample of 5,888. Race was determined by self-identification at interview. In addition to the 5 categories used in the ARIC study, participants were also asked a second question as to whether they considered themselves to be of Hispanic origin. To reduce the possibility of confounding by population structure, these analyses were limited to participants of self-described European-ancestry. The study sample for these analyses includes participants who were free of CVD at baseline, had blood samples drawn at their baseline examination, consented to genetic testing, had DNA available, and had successful genome-wide genotyping assay.

Participants were examined annually from enrollment to 1999, and since then continue to be under surveillance for stroke. Since baseline, participants have also been contacted twice a year to identify potential cardiovascular events, including stroke. In addition, all hospitalizations were screened for potential stroke events. For suspected events, information was collected from the participant or next of kin, from medical records, and, if needed, from the participant's physician. When available, CT and/or MRI scans or reports were reviewed centrally. Final adjudication of the occurrence of stroke, stroke types, and subtypes was undertaken by vascular neurologists at a consensus conference using all available information.

Strokes were classified as ischemic if there was imaging (CT or MRI within 4 weeks), surgical or autopsy evidence excluding a hemorrhage, or in the absence of such direct evidence (in <10% of cases in FHS and Rotterdam, none in CHS) if the preponderance of indirect evidence (e.g. deficit limited to one limb or completely resolved within 72 hours, atrial fibrillation in persons not on anticoagulants) suggested the event was an ischemic rather than a hemorrhagic stroke. A stroke was classified as hemorrhagic if there was imaging, surgical, lumbar puncture or autopsy evidence of hemorrhage, and in the absence of direct evidence to the contrary, when the participant lost consciousness permanently or died within hours after onset of focal signs. The stroke type was defined as unknown if there was insufficient information available to categorize the event as ischemic or hemorrhagic. All ischemic and hemorrhagic strokes and strokes of unknown type were included in the analyses of total stroke with one exception: subarachnoid hemorrhages (n=28 across all studies) were excluded from these analyses since the heritability, risk factors and pathophysiologic mechanisms underlying subarachnoid hemorrhages are distinctly different from other stroke subtypes. Persons with a subarachnoid hemorrhage were censored at the time of the event.

Only known ischemic strokes were included in the analysis of ischemic stroke. In secondary analyses we related those SNPs which reached genome-wide significance in our initial GWAS to the specific ischemic stroke subtype of atherothrombotic stroke, also called atherothrombotic brain infarction (ABI). We used the best available definitions of definite and possible ABI in each cohort; both large artery atherosclerotic strokes and small-vessel or lacunar strokes were included in this phenotype,

events known to be cardioembolic were excluded. For the analysis of ABI, participants were censored when they developed an alternative type of stroke.

Framingham Heart Study (FHS)

The FHS is a three-generation, single-site, community-based, ongoing cohort study that was initiated in 1948 to investigate prospectively the risk factors for CVD including stroke. It now comprises 3 generations of participants (N=10,333): the Original cohort followed since 1948;[7] their Offspring and spouses of the Offspring, followed since 1971;[8] and children from the largest Offspring families enrolled in 2000 (Gen 3).[9] Gen 3 participants were not included in this analysis since they are young (mean age 40±9 years) and few have suffered strokes. The Original cohort enrolled 5209 men and women who comprised two-thirds of the adult population then residing in Framingham, MA. Survivors continue to receive biennial examinations. The Offspring cohort comprises 5124 persons (including 3514 biological offspring) who have been examined approximately once every 4 years. The population of Framingham was virtually entirely white (Europeans of English, Scots, Irish and Italian descent) in 1948 when the Original cohort was recruited. At the initial examination participants were asked for country of birth and whether or not they had any Italian ancestry. At a later examination (the 8th) the Offspring cohort participants were asked to identify their race from the following choices: Caucasian or white, African-American or black, Asian, Native Hawaiian or other Pacific Islander, American Indian or Alaska native or 'prefer not to answer'. They were also asked to identify their ethnicity as either 'Hispanic or Latino' or neither. Almost all the FHS Original and Offspring participants are white/Caucasian and none were excluded from the discovery cohort.

At each clinic exam, participants receive questionnaires, physical examinations and laboratory testing; between examinations they remain under surveillance (regardless of whether or not they live in the vicinity) via physician referrals, record linkage and annual telephone health history updates. Incident strokes have been identified since 1948 through this ongoing system of FHS clinic and local hospital surveillance and methods used have been detailed previously;[10-12] they include review of medical records and collaboration with local general practitioners, emergency rooms and imaging facilities. If a participant saw a physician or was admitted to the hospital, visited an emergency room or obtained any brain imaging between biennial examinations for symptoms suggestive of TIA or stroke, a stroke neurologist from the Heart Study attempted to visit the person within 48 hours and recorded a complete history and neurological examination; this was repeated at 1, 3 and 6 months. All medical records from practitioners, hospitals, imaging centers, rehabilitation centers and nursing homes were procured for review. A panel of 3 investigators (at least 2 neurologists) adjudicated the diagnosis of stroke and determined stroke subtype in each case based on the Framingham evaluations and external records. The recruitment of Original and Offspring cohort participants at FHS had occurred long before the DNA collection with the result that a large number of stroke events in the FHS (although ascertained prospectively) were prevalent at the time of DNA collection and were excluded from these analyses. While this reduced the sample size from FHS, the meta-analyses presented here focused on incident events.

Strokes were classified as ischemic if there was imaging (CT or MRI within 4 weeks), surgical or autopsy evidence excluding a hemorrhage, or in the absence of such direct evidence (in <10% of cases in FHS and Rotterdam, none in CHS) if the preponderance of indirect evidence (e.g. deficit limited to one limb or completely resolved within 72 hours, atrial fibrillation in persons not on anticoagulants) suggested the event was an ischemic rather than a hemorrhagic stroke. A stroke was classified as hemorrhagic if there was imaging, surgical, lumbar puncture or autopsy evidence of hemorrhage, and in the absence of direct evidence to the contrary, when the participant lost consciousness permanently or died within hours after onset of focal signs. The stroke type was defined as unknown if there was insufficient information available to categorize the event as ischemic or hemorrhagic. All ischemic and hemorrhagic strokes and strokes of unknown type were included in the analyses of total stroke with one exception: subarachnoid hemorrhages (n=28 across all studies) were excluded from these analyses since the heritability, risk factors and pathophysiologic mechanisms underlying subarachnoid hemorrhages are distinctly different from other stroke subtypes. Persons with a subarachnoid hemorrhage were censored at the time of the event.

Only known ischemic strokes were included in the analysis of ischemic stroke. In secondary analyses we related those SNPs which reached genome-wide significance in our initial GWAS to the specific ischemic stroke subtype of atherothrombotic stroke, also called atherothrombotic brain infarction (ABI). We used the best available definitions of definite and possible ABI in each cohort; both large artery atherosclerotic strokes and small-vessel or lacunar strokes were included in this phenotype,

events known to be cardioembolic were excluded. For the analysis of ABI, participants were censored when they developed an alternative type of stroke. A lacunar stroke was diagnosed based on either clinical presentation of a typical lacunar syndrome, or a clinical picture compatible with a lacunar lesion and imaging showing a small ischemic lesion in the territory of the deep penetrating arteries. An ischemic stroke was categorized as cardioembolic if there was evidence of a cardiac or aortic source of embolization based on the clinical presentation, brain imaging and review of all available cardiac assessments including ECG, echocardiography and cardiac monitoring.

deCODE Genetics

Cases, irrespective of age, were identified from a registry of individuals diagnosed with ischemic stroke or TIA at Landspitali University Hospital in Reykjavik, the only tertiary referral centre in Iceland, during the years 1993 to 2006. The ischemic stroke or TIA diagnoses were based on standard WHO criteria and imaging evidence (either CT or MRI), and were clinically confirmed by neurologists. Eligible patients who survived the stroke were invited to participate the genetic study, either by attending a recruitment centre for deCODE's genetic studies, or they were visited at their home by a study nurse. Control subjects were participants from a large variety of genetic programs at deCODE. Individuals with confirmed stroke (identified by cross-matching with hospital lists), who had participated in genetic studies other than those of cardiovascular diseases (CVD) (but not participated in CVD studies) were excluded as controls.

The Genetics of Early Onset Stroke (GEOS) Study, Baltimore, USA

GEOS is a population-based case-control study designed to identify genes associated with early-onset stroke in patients with first-ever ischemic stroke aged 15-49 years from the greater Baltimore-Washington area between 1992 and 2008. Only patients of European descent are included in this meta-analysis. Cases were identified through discharge surveillance from 59 participating hospitals and direct physician referral from a defined geographic region. Abstracted medical records were reviewed and adjudicated for ischemic stroke subtype by two neurologists, with discrepancies resolved by a third neurologist. Controls with no history of ischemic stroke were identified through random digit dialing and were frequency-matched to cases based on sex, age, geographic location and, during the later study periods, ethnicity.

Heart Protection Study (HPS)

The Heart Protection Study (HPS) was a large randomized trial involving individuals at increased risk of vascular events. Between 1994-1997 20,536 men and women aged 40-80 years were recruited from 69 collaborating hospitals in the United Kingdom (with ethics committee approval). Participants were eligible for inclusion provided they had non-fasting blood total cholesterol concentrations of at least 135 mg/dL (3.5 mmol/L) and either a previous diagnosis of coronary disease, ischemic stroke, other occlusive disease of non-coronary arteries, diabetes mellitus, or (if were men 65 years or older) treated hypertension. None of them was on statin therapy. At the initial screening visit, all participants provided written consent and began a "run-in" phase involving 4 weeks of placebo followed by 4 to 6 weeks of 40 mg simvastatin daily, after which compliant and eligible individuals were randomly allocated 40 mg simvastatin daily or matching placebo for approximately 5 years. Individuals entering HPS with a clinical diagnosis of ischemic stroke were used as cases in the METASTROKE study. Individuals entering HPS with pre-existing diabetes but no history of cerebrovascular disease, coronary heart disease or peripheral vascular disease were used as controls.

The Heart and Vascular Health Study (HVH)

The setting for this study was Group Health (GH), a large integrated health care system in western Washington State. Data were utilized from an ongoing case-control study of incident myocardial infarction (MI) and stroke cases with a shared common control group. Methods for the study have been described previously [13-15] and are briefly summarized below. The study was approved by the human subjects committee at GH, and written informed consent was provided by all study participants.

All study participants were GH members and aged 30-79 years. MI and stroke cases were identified from hospital discharge diagnosis codes and were validated by medical record review. Controls were a random sample of GH members frequency matched to MI cases on age (within decade), sex, treated hypertension, and calendar year of identification. The index date for controls was a computer-generated random date within the calendar year for which they had been selected. For stroke cases, the index date was the date of admission for the first acute stroke. Participants were excluded if they were recent enrolees at GH, had a history of prior stroke, or if the incident event was a complication of a procedure or surgery.

Trained medical record abstractors collected eligibility and risk factor information from a review of the GH medical record using only data available prior to the index date and through a telephone interview. Medication use was ascertained using computerized GH pharmacy records. A venous blood sample was collected from all consenting subjects, and DNA was extracted from white blood cells using standard procedures.

Diagnostic criteria for ischemic stroke were adopted from the Cardiovascular Health Study.[16] These criteria included (1) rapid onset of neurologic deficit or subarachnoid hemorrhage, (2) deficit persisting for longer than 24 hours unless computed tomography or magnetic resonance imaging show evidence of permanent damage, and (3) no underlying brain trauma, tumor, or infection to cause symptoms.

These analyses were limited to ischemic stroke cases, namely those satisfying 1 or more of the following criteria: (a) Focal deficit, without evidence of blood on CT or MRI, (b) Focal deficit, with mottled appearance in the appropriate location on CT, **or** (c) surgery or autopsy evidence of infarction.

Among ischemic strokes, the subtypes were defined as follows:

Lacunar stroke ("SVD") required either: (a) CT/MRI demonstrates a deep area of infarction (decreased density) less than 2 cm. across, or (b) A normal CT, but the clinical syndrome is typical of a lacunar infarction, that is: a pure motor stroke, a pure sensory stroke, hemiparesis plus ataxia, or dysarthria plus a clumsy hand. Embolic stroke ("CE") required either (a) a recognized source of emboli such as atrial fibrillation, endocarditis, mitral stenosis, thrombus in heart, recent MI or cardiac surgery, or (b) a mottled appearance consistent with infarction on the CT. Atherosclerotic infarction ("LAA") when there is no apparent source of emboli or evidence of lacunar infarction and there is evidence of large vessel atherosclerosis by carotid ultrasound or angiography.

The Ischemic Stroke Genetics Study (ISGS)/ Siblings With Ischemic Stroke Study (SWISS)
The Siblings with Ischemic Stroke Study (SWISS) is a multicenter affected sibling pair study enrolling probands with ischemic stroke at 66 US medical centers and 4 Canadian medical centers.[17] All probands are adult men and women over the age of 18 years diagnosed with ischemic stroke confirmed by a study neurologist on the basis of history, physical examination and CT or MR imaging of the brain. Additionally all probands were required to have at least one living sibling with a history of stroke. Siblings were enrolled using proband-initiated contact or direct contact when permitted by Institutional Review Boards.[18] Clinical exclusion criteria mirrored that in ISGS. Concordant siblings had their diagnosis of ischemic stroke confirmed by review of medical records by a central vascular neurology committee. Subtype diagnoses were assigned to the index strokes of probands and concordant siblings according to TOAST criteria.[19]
Readily available US controls were utilized, including stroke-free participants from the Baltimore Longitudinal Study of Aging and the National Institute of Neurological Diseases and Stroke neurologically normal control series taken from the Coriell Cell Repositories. All controls had been previously genotyped and described in detail elsewhere.

The Ischemic Stroke Genetic Study (ISGS) is a multicenter study where inpatient cases were recruited from five United States academic medical centers.[20] Cases are adult men and women over the age of 18 years diagnosed with first-ever ischemic stroke confirmed by a study neurologist on the basis of history, physical examination and CT or MR imaging of the brain who were enrolled within 30 days of onset of stroke symptoms. Cases exclusion criteria include: a mechanical aortic or mitral valve at the time of the index ischemic stroke, central nervous system vasculitis, bacterial endocarditis, cerebral autosomal dominant arteriopathy with subcortical infarcts and leukoencephalopathy (CADASIL), Fabry disease, homocystinuria, mitochondrial encephalopathy with lactic acidosis and stroke-like episodes (MELAS), or sickle cell anemia. Stroke severity at enrollment was assessed using the NIH Stroke Scale with the diagnostic evaluation including head CT (95%) or MRI (83%), electrocardiography (92%), cervical arterial imaging (86%), and echocardiography (74%). Medical records from all cases were centrally reviewed by a vascular neurology committee and assigned ischemic stroke subtype diagnoses according to criteria from the Trial of ORG10172 (TOAST), the Oxfordshire Community Stroke Project, and the Baltimore-Washington Young Stroke Study.

The MGH Genes Affecting Stroke Risk and Outcome Study (MGH-GASROS)
Cases were all consecutive patients aged ≥ 18 years presenting with ischemic stroke and admitted to the Massachusetts General Hospital (MGH) Stroke Unit through the Emergency Department, or

evaluated in the MGH Neurology outpatient clinics, as well as on the inpatient Medical and Vascular Surgical services from January 2003 to July 2008. Only patients of European ancestry (confirmed by principal component analysis using genome-wide SNP data) were included in the present analysis. Ischemic stroke was defined as either (1) a radiographically proven (head CT or MRI) infarct associated with the appropriate clinical stroke syndrome, or (2) a fixed neurological deficit persisting more than 24 hours, consistent with a vascular pattern of involvement and without radiographic evidence of demyelinating disease, or other non-vascular structural disease. Patients with specific vascular disorders (vasculitis, subacute bacterial endocarditis, fibromuscular dysplasia, vasospasm) were excluded from the study. All subjects were evaluated by a neurologist upon presentation and provided informed consent. Clinical and laboratory data were collected during the admission for qualifying ischemic stroke event. Diagnostic work-up included: head CT (100%), brain MRI (90%), cervical and intracranial vessel imaging using CTA or MRA (75%), carotid and/or transcranial ultrasound (24%), echocardiography (86%), and Holter monitoring (16%). Controls were recruited among the stroke-free adults presenting to the MGH outpatient clinics and matched with the stroke cases on the basis of age, sex and ancestry information obtained from principal component analysis of GWAS data.

Milano

This study Includes consecutive Italian patients referred to Besta Institute from 2000 to 2009 with stroke and included in the Besta Cerebrovascular Diseases Registry (CEDIR). Ischemic stroke cases, first ever or recurrent, confirmed on brain imaging, were selected for this study. All cases were of self reported Caucasian ancestry and had clinically relevant diagnostic workup performed. All cases were phenotyped by an experienced stroke neurologist according to TOAST criteria, based on relevant clinical imaging and available information on cardiovascular risk factors. Controls are Italian individuals enrolled within the PROCARDIS Study, with no personal or sibling history of coronary heart disease before age 66 years.

Rotterdam

The Rotterdam Study is a population-based cohort study among inhabitants of a district of Rotterdam (Ommoord), The Netherlands, and aims to examine the determinants of disease and health in the elderly with a focus on neurogeriatric, cardiovascular, bone, and eye disease.[21] In 1990-1993, 7,983 persons participated and were re-examined every 3 to 4 years.

After enrollment in the Rotterdam Study, participants are continuously monitored for incident stroke through automated linkage of the study database with files from general practitioners. Nursing home physicians' files and files from general practitioners of participants who moved out of the district are scrutinized as well. Additional information is obtained from hospital records. Potential strokes are reviewed by research physicians and verified by an experienced stroke neurologist. Stroke is defined as rapidly developing clinical signs of focal or global disturbance of cerebral function with no apparent cause other than a vascular origin. History of stroke at baseline was assessed during the baseline interview and verified in medical records. Strokes are further classified as cerebral infarction or intracerebral hemorrhage based on neuroimaging reports. If neuroimaging is lacking, a stroke is classified as unspecified. Subarachnoid hemorrhages were excluded. Ischemic strokes are subtyped into large-vessel disease, small vessel disease, or cardio-embolic based on all available clinical and imaging data. If insufficient information is present to reliably subtype into one of three categories, the ischemic stroke is classified as unspecified ischemic stroke. For the current study, too few cases of ischemic stroke subtypes were present to reliably perform statistical analyses.

Participants were followed from baseline to stroke, death, last health status update when they were known to be stroke-free, or January 1, 2005, whichever came first. Follow-up was complete up to January 1, 2005, for 99.1% of potential person-years.

Wellcome Trust Case-Control Consortium 2 (WTCCC2)

The WTCCC2 samples were genotyped as part of the WTCCC 2 ischemic stroke study. Stroke cases included samples recruited by investigators at St. George's University London (SGUL) and University of Oxford in the UK and the Department of Neurology, Klinikum Großhadern, Ludwig-Maximilians-University, Munich.

The SGUL collection comprised 1224 ischemic stroke samples from a hospital based setting. All cases were of self reported Caucasian ancestry. Ischemic stroke subtypes were determined according to TOAST criteria based on relevant clinical imaging and available information on cardiovascular risk

factors. The University of Oxford collection comprised 896 ischemic stroke cases, consecutively collected as part of the Oxford vascular study (OXVASC). Cases were of self reported Caucasian ancestry, and ischemic stroke subtypes were determined according to TOAST criteria based on relevant clinical imaging. The University of Edinburgh collection comprised 727 ischaemic stroke cass, consecutively collected as part of the Edinburgh Stroke Study. Cases were of self-reported Caucasian ancestry, with ischaemic stroke subtypes determined according to TOAST criteria based on relevant clinical and imaging data. The Munich samples included 1383 ischemic stroke cases. Cases were consecutive European Caucasians recruited from a single dedicated Stroke Unit at the Department of Neurology, Klinikum Großhadern, Ludwig-Maximilians-University, Munich. Ischemic stroke subtypes were determined according to TOAST criteria based on relevant clinical and imaging data.

Controls for the UK samples were drawn from shared WTCCC controls obtained from the 1958 Birth Cohort. This is a prospectively collected cohort of individuals born in 1958 (http://www.b58cgene.sgul.ac.uk/), and ascertained as part of the national child development study (http://www.cls.ioe.ac.uk/studies.asp?section=000100020003). Data from this cohort are available as a common control set for a number of genetic and epidemiological studies. For the German samples controls were Caucasians of German origin participating into the population KORAgen study (www.gsf.de/kora/en/english.html). This survey represents a gender- and age stratified random sample of all German residents of the Augsburg area and consists of individuals 25 to 74 years of age, with about 300 subjects for each 10-year increment. All controls were free of a history of stroke or transient ischemic attack.

Genome-wide genotyping and quality control in discovery studies

The Atherosclerosis Risk in Communities Study (ARIC)
Methods of genotyping, quality-control assessment and filtering, and genotype imputation have been described in [3]. Briefly, genotyping was performed with the GeneChip SNP Array 6.0 (Affymetrix). Subject specific quality control filters included filters for call rate, heterozygosity, sex mismatch. SNP specific quality control filters included filters for call rate, minor allele frequency (MAF), Hardy-Weinberg equilibrium (HWE), and differential missingness by outcome or genotype. The set of genotyped input SNPs used for imputation was selected based on high quality GWA data. We used a callrate >95%, HWE p-value>$5x10^{-6}$; MAF>0.01. A total of 704,588 SNPs passing QC criteria were used for imputation, which was performed with the MaCH v1.0.16 software.

Australian Stroke Genetics Collaborative (ASGC)
The ASGC sample was genotyped using the Illumina HumanHap610-Quad array. Quality control excluded SNPs with genotype call rate <0.95, deviation from Hardy-Weinberg equilibrium ($P<1\times10^{-6}$) or minor allele frequency <0.01. At the sample level, quality control excluded individuals with: (i) genotype call rate <95% (n=4); ii) genome-wide heterozygosity < 23.3% or > 27.2% (n=9); iii) inadequate clinical data or inconsistent clinical and genotypic gender (n=45) and; iv) an inferred first- or second-degree relative in the sample based on pair-wise allele sharing estimates (estimated genome proportion shared identical by descent (IBD): pi-hat >0.1875 : *n*=37). Following these exclusions, Eigenstrat principal components analysis (PCA) was performed, incorporating genotype data from Phase 3 HapMap populations (CEU, CHB, JPT, TSI, YRI). In eigenvector plots, the majority of ASGC samples clustered closely with European (CEU and TSI) reference populations. Eighteen samples (16 cases and 2 controls) showed prominent evidence of Asian ancestry and were removed. Principal component and IBD analyses were performed using a pruned subset of quasi-independent SNPs (~130,000 SNPs) to avoid confounding by linkage disequilibrium (LD). Following quality control, 1162 cases and 1244 controls were available for association analyses at 551,514 SNPs.

Genotype imputation in the filtered sample was performed using MACH v1.0.16 (http://www.sph.umich.edu/csg/yli/mach/index.html), based on HapMap Phase 2 (release #24) phased haplotypes for European-ancestry (CEU) samples. Subsequent quality control excluded imputed SNPs with MAF <0.01 or ratio of observed dosage variance to expected binomial variance of r^2 <0.3.

Bio-Repository of DNA in Stroke (BRAINS)
The BRAINS sample was genotyped using the Illumina HumanHap610-Quad array. Quality control excluded SNPs not genotyped on all case and control collections and SNPs with genotype call rate <0.95, deviation from Hardy-Weinberg equilibrium ($P<1\times10^{-6}$) or minor allele frequency <0.01.

Individual samples were excluded due to low call rates (<95%), gender discrepancy, unexpected relatedness or evidence of non-European ancestry.

Genotype imputation was performed using MACH v1.0.16 (http://www.sph.umich.edu/csg/yli/mach/index.html), based on HapMap Phase 2 (release #22) phased haplotypes for European-ancestry (CEU) samples. Quality control removed imputed SNPs with MAF <0.01 or ratio of observed dosage variance to expected binomial variance of r^2 <0.3. Analyses were performed using PLINK v 1.6 (http://pngu.mgh.harvard.edu/~purcell/plink/).

Cardiovascular Health Study (CHS)
In 2007-2008, genotyping was performed at the General Clinical ResearchCenter's Phenotyping/Genotyping Laboratory at Cedars-Sinai using the Illumina 370CNV Duo® BeadChip system on the first 2,427 of 3,980 CHS participants who were free of CVD at baseline. The 1,908 persons excluded for prevalent CVD had prevalent coronary heart disease (n=1195), congestive heart failure (n=86), peripheral vascular disease (n=93), valvular heart disease (n=20), stroke (n=166) or transient ischemic attack (n=56). Some persons had more than one reason to be excluded and for these individuals only the initial exclusionary event is listed. The 2,427 participants who had been genotyped by July 2008(time of the meta-analysis) were a stratified probability sample that included all cases of myocardial infarction (MI), stroke, atrial fibrillation, dementia, and heart failure, and a selection of controls sampled based on the age-, and sex distribution of MI cases. Sampling weights were used in the analysis to account for the study design and to weight back to the underlying cohort selected for genotyping (N=3,980). Because the other cohorts were predominantly white, the African American participants were excluded from the initial meta-analysis to limit the potential for false positive associations due to population stratification. At the time of the meta-analysis, genotyping had been attempted in 2,101 white participants, and was successful in 2,022 persons; the latter constitute the CHS sample for this study. QC criteria used to define successful genotyping are listed elsewhere, 35 persons were excluded for a subject specific call rate ≤95%.

The set of genotyped input SNPs used for imputation was selected based on the highest quality GWA data. We used a callrate >95%; a minor allele frequency >0.01; a Hardy- Weinberg p>1x10-9; and a test of differential missingness by the "mishap" test in PLINK p>1x10-9. We used BIM-BAM to impute to the plus strand of NCBI build 35. For each imputed SNP a reliability of imputation was estimated (as the ratio of the empirically observed dosage variance to the expected binomial dosage variance: O/E ratio).

deCODE Genetics
The Icelandic chip-genotyped samples were assayed with the Illumina Human Hap300, Hap CNV370, Hap 610, 1M or Omni-1 Quad bead chips at deCODE genetics. SNPs were excluded if (i) yield was lower than 95%, (ii) minor allele frequency was less than 1% in the population, (iii) significant deviation from Hardy-Weinberg equilibrium was observed in the controls (P < 0.001), (iv) an excessive inheritance error rate (over 0.001) was produced or (v) there was a substantial difference in allele frequency between chip types (from just a single chip if that resolved all differences, but from all chips, otherwise). All samples with a call rate below 97% were excluded from the analysis. Imputation was performed using IMPUTE.

Framingham Heart Study (FHS)
FHS participants had DNA extracted and provided consent for genotyping in the 1990s. All available eligible participants were genotyped at Affymetrix (Santa Clara, CA) through an NHLBI funded SNP-Health Association Resource (SHARe) project using the Affymetrix GeneChip® Human Mapping 500K Array Set and 50K Human Gene Focused Panel.® In 272 persons (31 with stroke), small amounts of DNA were extracted from stored whole blood and required whole genome amplification prior to genotyping. Cell lines were available for most of the remaining participants. Genotyping was attempted in 5293 participants, and 4,519 persons met QC criteria. Failures (call rate <97%, extreme heterozygosity or high Mendelian error rate) were largely restricted to persons with whole-genome amplified DNA and DNA extracted from stored serum samples. We also excluded 156 participants who were less than 45 years old at the time of the DNA draw, 135 persons with prevalent stroke and 97 persons who did not have stroke surveillance on follow-up after genotyping; the remaining 4,131 subjects constitute the FHS sample for this study.

The set of genotyped input SNPs used for imputation was selected based on the highest quality GWA data. We used a callrat; >97%; a minor allele frequency >0.01; a Hardy- Weinberg p>1x10-6; and a

test of differential missingness by the "mishap" test in PLINK p>1x10-9 in each study. We used the Markov Chain Haplotyping (MaCH) package (http://www.sph.umich.edu/csg/abecasis/MACH) version 1.0.15 software to impute to plus strand of NCBI build 36, HapMap release #22. For each imputed SNP a reliability of imputation was estimated (as the ratio of the empirically observed dosage variance to the expected binomial dosage variance: O/E ratio). For the primary meta-analysis using inverse-variance weighting less weight is given to imputed SNPs with low observed dosage variance (resulting in higher variance of the estimate). For the secondary meta- analysis using the inverse square root (N) weighting the ratio was used to compute an effective sample size.

The Genetics of Early Onset Stroke (GEOS) Study, Baltimore, USA
Samples were genotyped at the Johns Hopkins Center for Inherited Disease Research (CIDR), and genotyping was performed using the Illumina HumanOmni1-Quad_v1-0_B BeadChip (Illumina, San Diego, CA, USA). Case and control samples were balanced across the plates. Allele cluster definitions for each SNP were determined using Illumina BeadStudio Genotyping Module version 3.3.7, Gentrain version 1.0 and the combined intensity data from all released samples. Genotypes were not called if the quality threshold (Gencall score) was below 0.15.

All samples had a genotype call rate > 98%. Genotyping concordance rate was 99.996% based on study duplicates. Samples were excluded due to unexpected duplicates, gender discrepancy, unexpected relatedness or evidence of non-European ancestry based on principal components analysis. Individual SNPs were excluded from analysis if they had excessive deviation from Hardy-Weinberg Equilibrium (HWE) proportions ($P < 1.0x10^{-6}$), genotype call rates <97.5% or minor allele frequency < 1%. Departure from HWE was assessed by chi-square test among controls only.

Heart Protection Study (HPS)
DNA was extracted from stored white cells and genotyping was carried out at the Centre National de Génotypage in Evry, France. Genotypes were measured using the Illumina 610K Quad panel, called using Illumina BeadStudio software, and imputed with reference to HapMap2 CEU release 22 (build 36) using MACH. Single nucleotide polymorphisms with <97.5% call rate, significant deviation from Hardy-Weinberg equilibrium ($p<1x10^{-6}$) or low minor allele frequency (<0.01) were excluded. Genotype data were available for 578 stroke cases and 468 controls after quality control exclusions for discrepant sex, repeated samples and non-European ancestry. Statistical analyses were performed using MACH2QTL.

The Heart and Vascular Health Study (HVH)
Genotyping was performed at the General Clinical Research Center's Phenotyping/Genotyping Laboratory at Cedars-Sinai using the Illumina 370CNV BeadChip system. Genotypes were called using the Illumina BeadStudio software. Samples were excluded from analysis for sex mismatch or call rate < 95%. The following exclusions were applied to identify a final set of 301,321 autosomal SNPs: call rate < 97%, HWE $P < 10^{-5}$, > 2 duplicate errors or Mendelian inconsistencies (for reference CEPH trios), heterozygote frequency = 0, SNP not found in HapMap, inconsistencies across genotyping batches. Imputation was performed using BIMBAM with reference to HapMap CEU using release 22, build 36 using one round of imputations and the default expectation-maximization warm-ups and runs.

Logistic regression was used to investigate the association of each SNP with the risk of stroke and MI, adjusting for the matching factors of age, sex, hypertension status and index year. We used linear additive models with robust standard errors and estimated risk for each additional copy of the variant allele, using R. SNPs were excluded from analysis for variance on the allele dosage ≤0.01.

The Ischemic Stroke Genetics Study (ISGS)/ Siblings With Ischemic Stroke Study (SWISS)
Both the ISGS/SWISS cases were genotyped using the Illumina 610 or 660 genotyping arrays, while control series used in the ISGS/SWISS dataset were genotyped using the Illumina HumanHap 550Kv1 or 550Kv3 genotyping arrays. Genotypes were called using Illumina GenomeStudio software, with all alleles called on the forward strands. All A/T and G/C SNPs were removed prior to merging case and control sample sets. Also SNPs with discordant minor alleles on the same strand across microarrays were removed prior to merging datasets. Preliminary exclusion criteria per sample included genome-wide SNP call rates <95% and discordance between self-reported gender and sex determined from X chromosome heterozygosity. SNPs were excluded from the merged case-control series if genotyping success rate <95%, minor allele frequency (MAF) <0.01, Hardy-Weinberg equilibrium (HWE) P<1E−4 in controls and P<1E−7 in cases, nonrandom missingness per haplotype P<1E−5 and missingness in cases compared to controls (from chi-squared test) P<1E−5.

ISGS/SWISS cases and controls were merged with a subset of samples from HapMap 3 (ASW, CEU, CHB, JPT, TSI and YRI populations) and underwent multidimensional scaling analyses to verify European ancestry with principal component vector 1 (PC1) and 2 (PC2) values greater than 3 standard deviations from the combined CEU/TSI means for each vector were excluded as outliers. Samples were excluded if they shared greater than a 0.125 proportion of alleles (pi_hat >0.125). Basic quality control of genotyped SNP data was carried out using PLINKv1.07. The SNPs passing quality control for the ISGS/SWISS were imputed using a two-stage procedure implemented in Markov Chain based haplotyper (MACH; version 1.0.16) under default settings. For this study, the August 2010 release of the 1000 Genomes European ancestry haplotypes was utilized as a reference for SNP imputation.

The MGH Genes Affecting Stroke Risk and Outcome Study (MGH-GASROS)
Cases and controls were genotyped using the Affy 6.0 array. Quality control procedures excluded SNPs with >5% missingness, minor allele frequency <0.01, or Hardy-Weinberg p-value < 10^{-7}. Individual samples were excluded if they exhibited genotype missingness >5%, cryptic relatedness (one of each pair demonstrating pi_hat [estimated proportion of genome shared identical by descent] > 0.15 was removed), or non-European ancestry based on multi-dimensional scaling analysis using HapMap Phase 3 populations. Analyses were performed using PLINK v 1.6 (http://pngu.mgh.harvard.edu/~purcell/plink/). Imputation was performed using MACH v 1.0.16 (http://www.sph.umich.edu/csg/yli/mach/index.html) and the HapMap 3 CEU+TSI training set.

Milano
Italian cases were genotyped using Illumina Human610-Quad v1_B or Human660W-Quad v1_A chips. Italian controls were genotyped with the Illumina HumanHap610-Quad chip. PCA with HapMap 3 on the Italian cases showed that Italian PROCARDIS controls had similar ancestry to the cases. All samples had a genotype call rate > 95%. Samples were excluded due to unexpected duplicates or evidence of non-European ancestry based on principal components analysis. Quality control procedures excluded SNPs with MAF <0.01 or Hardy-Weinberg P-value <5×10-6 in either the case or control collections.

Rotterdam
All participants had DNA extracted at baseline in 1990-1993. In 2007-2008, genotyping was attempted in participants with high-quality extracted DNA at baseline. Genotyping was done at the Human Genotyping Facility, Genetic Laboratory Department of Internal Medicine, Erasmus MC, Rotterdam, The Netherlands. Genotyping was carried out using the Illumina HumanHap550 Duo BeadChip® according to the manufacturer's protocols. Participant-specific quality-control included filters for call rate, heterozygosity, and number of Mendelian errors per individual. SNP-specific quality control included filters for call rate, minor allele frequency, Hardy-Weinberg equilibrium, and differential missingness by outcome or genotypes (mishap test in PLINK). Data were screened for latent substructure, including cryptic relatedness, using IBD matrix.

The set of genotyped input SNPs used for imputation was selected based on their highest quality GWA data. We used a call rate >98%, a minor allele frequency >0.01, a Hardy-Weinberg p>$1x10^{-6}$ Rotterdam, and a test of differential missingness by the "mishap" test in PLINK p>$1x10^{-9}$ to selected SNPs used for imputation. We used the Markov Chain Haplotyping (MaCH) package (http://www.sph.umich.edu/csg/abecasis/MACH) to impute to the plus strand of NCBI build 36, HapMap release #22. Imputation of genotypes provides a dosage value for every SNP between 0 and 2 indicating the expected value of a SNP being homozygous for the reference allele. For each imputed SNP an assessment of the informativeness of the imputation was estimated (as the ratio of the empirically observed dosage variance to the expected binomial dosage variance, O/E ratio).

Wellcome Trust Case-Control Consortium 2 (WTCCC2)
All WTCCC2 cases were genotyped as part of the WTCCC2 Ischemic Stroke study using the Illumina Human660W-Quad array. British controls were genotyped using the Illumina Human1.2M-Duo. German controls were genotyped on the Illumina Human 550k platform. Quality control procedures in the WTCCC2 excluded SNPs not genotyped on all case and control collections and SNPs with Fisher information measure <0.98, genotype call rate <0.95, MAF <0.01 or Hardy-Weinberg P-value <1×10-20 in either the case or control collections. Samples were excluded if identified as outliers on call rate, heterozygosity, ancestry and average probe intensity based on a Bayesian clustering algorithm. Samples were also removed if they exhibited discrepancies between inferred and recorded gender or cryptic relatedness with other WTCCC2 samples (pairwise identity-by-descent >0.05). Autosomal

genotype imputation was performed using MACH based on HapMap Phase 2 European (CEU) reference data.

Supplementary Table 6: Proportion of stroke cases with investigations performed in the replication cohorts.

Cohort	Number of Cases	Brain imaging with CT or MRI (%)	MRI (%)	ECG (%)	Echocardiographic Imaging (%)	Extracranial cerebral artery imaging (%)
Barcelona	439	100	18	100	70	100
BSS	225	100	-	100	100	100
Copenhagen	730	-	-	-	-	-
Go-Darts***	737	-	-	-	-	-
ESS	276	100	21	99	33	95
Glasgow	675	100	18	100	35	100
Graz	657	100	41.9	99.7	39.2	99.7
Interstroke*	1126	99.3	14.2	98.8	37.6	37.5
Krakow	1235	100	8.0	100	54.8	85.3
Leuven*	531	100	89	100	95	99.6
Lund	424	100	-	100	17.2	-
Munster	1232	97.2	45.4	-	77.4	97.7
Portugal	539	100	25	97	-	-
Pakistan (RACE)	1322	100	55	100	100	91
SMART	623	90.7	-	-**	-	100
Swedish (Karolinska)	876	100	16	100	38	-
VISP*	1726	99.9	47.0	99.9	-	-
WHI	302	-	-	-	-	-

BSS=Belgium Stroke Study. ESS=Edinburgh Stroke Study. Go-Darts=4G5enetics of Diabetes Audit and Research in Tayside Study. RACE=Risk Assessment of Cerebrovascular Events Study, Pakistan. SMART=Second Manifestations of ARTerial disease. VISP=The Vitamin Intervention for Stroke Prevention Trial. WHI=The Women's Health Initiative. CT=Computed Tomography. MRI=Magnetic Resonance Imaging. ECG=Electrocardiogram. "-"=data not available, not recorded or performed only as clinically indicated. Extracranial cerebral arterial imaging includes carotid and vertebral artery ultrasound, or computed tomographic angiography, or magnetic resonance angiography. *=values given are for individuals prior to genotypic QC steps. **=We have no exact numbers of patients with ecg, probably 100% (not registered), but none of the included patients had atrial fibrillation; ***=Cases were identified through hospital admissions codes so while they may have had investigations performed we do not have that information for all cases.

Supplementary Table 7: Replication Cohort Characteristics

Sample	Sample size					Age (mean ± SD)					Male Gender (%)					CT/MRI Brain Imaging (%)
	IS	SVD	CE	LVD	controls	IS	SVD	CE	LVD	controls	IS	SVD	CE	LVD	controls	
Barcelona	439	150	179	110	404	72.0 ± 12.8	70.1± 12.2	75.4 ± 13.6	69.9 ± 11.3	71.5 ± 7.2	53.4	59.6	39.5	66.4	43.8	100
BSS	225	90	11	93	312	52.8 ± 5.4	53.5 ± 5.0	49.9 ± 8.5	52.4 ± 5.0	69.8± 8.8	64.0	66.0	36.4	64.5	64.5	99.6
Copenhagen	730	-	-	-	1545	63.1 ± 9.1	-	-	-	64.8 ± 10.7	45.5	-	-	-	62.6	-
ESS	276	69	40	20	987*	71.4 ± 11.4	67.4 ± 11.8	74.9 ± 13.1	75.1 ± 7.1	69.6 ± 0.83*	52.5	59.4	55.0	55.0	51.2*	100
Glasgow	675	150	125	91	987*	69.9 ± 12.2	69 ± 13.7	71.1± 10.3	68.5 ± 11.7	69.6 ± 0.83*	52.5	58.6	49.9	50	51.2*	100
Go-Darts	737	-	130	259	8442	69.0± 9.8	-	75.7 ± 8.6	70.9 ± 8.9	65.0 ± 11.4	59.2	-	61.0	61.6	51.2	-
Graz	657	207	116	108	848	68.9 ± 14.5	69.2 ± 15.6	72.7 ± 10.0	66.9 ± 13.7	65.2 ± 8.1	57.5	57.0	59.5	54.6	43.2	100
Interstroke*	872	238	143	198	926	62.6 ± 14.4	61.1± 13.5	67.9± 16.2	61.6± 14.6	61.2± 13.5	56.1	57.1	58.8	49.5	59.6	99.3
Krakow	1235	171	377	152	584	67.4±14.2	63.6 ± 12.3	70.1 ± 13.5	66.7 ± 11.2	58.4 ± 17.8	49.3	54.5	44.0	62.8	47.5	100
Leuven*	531	63	195	83	445	68.9 ± 13.5	65.6 ± 14.6	70.5 ± 13.0	68.7 ± 10.6	57.6 ± 14.2	60	62	61	67	43	100
Lund	424	94	140	21	466	74.4 ± 11.3	70.6 ± 12.1	78.1± 9.9	70.7 ± 9.3	73.4 ± 11.2	56.4	60.6	51.4	71.4	58.2	100
Munster	1232	224	478	528	1053	69.3 ± 14.5	68.4 ± 13.0	71.8± 14.5	67.5 ± 14.8	53.0±13.6	53.2	52.2	47.5	58.7	46.7	97.2
Portugal	539	-	-	-	507	52.5 ± 9.3	-	-	-	62.9 ± 6.9	63.1	-	-	-	46.2	100
RACE (Pakistan)	1322	189	225	195	1143	52.9 ± 10.6	53.8 ± 9.4	52.9 ± 11.2	54.5 ± 9.2	51.9 ± 7.9	52.4	52.4	62.2	50.8	53.4	100
SMART	623	195	30	368	6712	60.6 ± 10.9	58.8± 11.5	63.2± 10.6	61.7± 10.3	55.7± 12.5	68.9	70.8	60.0	68.5	68.2	90.7

Swedish (Karolinska)	876	75	157	177	742	69.0 ± 11.2	65.7 ± 10.1	72.4 ± 10.8	69.7 ± 9.2	43.1 ± 12.3	56.9	60.0	54.0	67.8	59.8	100
VISP	1726*	-	-	-	1047	68.0 ± 10.7	-	-	-	-	65.0	-	-	-	59.4	99.9
WHI	302	78	42	31	2099	-	-	-	-	-	0	0	0	0	0	-

BSS=Belgium Stroke Study. ESS=Edinburgh Stroke Study. Go-Darts=Genetics of Diabetes Audit and Research in Tayside Study. RACE=Risk Assessment of Cerebrovascular Events Study, Pakistan. SMART=Second Manifestations of ARTerial disease. VISP=The Vitamin Intervention for Stroke Prevention Trial. WHI=The Women's Health Initiative. IS=Ischaemic Stroke. SVD=small vessel disease. LVD=large vessel disease. CE=cardioembolic stroke. "-"=information not available or not relevant. *=values given are for individuals prior to genotypic QC steps.

Ascertainment and clinical assessment of participants in Replication studies

Barcelona
Patients with ischemic stroke were recruited consecutively at the stroke unit of Vall d'Hebron Hospital, Barcelona, Spain. Only patients admitted at the stroke unit with stroke resulting from large and small vessel disease as well as CE origin were included in the genetic study. All patients underwent brain imaging by CT scan, and some had additional MRI performed. ECG and extracranial and intracranial arterial ultrasound examination were performed in all patients, and Holter monitoring and echocardiography were performed on patients with clinical suspicion of a CE source or undetermined origin. Control participants were selected from relatives of patients (wife or husband, without any consanguinity among cases and controls) and healthy volunteers visiting the same hospital for routine testing. They were _65 years of age and classified as free of neurovascular and cardiovascular history and familial history of stroke by direct interview before recruitment.

The Belgian Stroke Study (BSS)
BSS is a case-control study. 237 stroke patients were classified according to the TOAST classification, all occurring between 45 and 60 years of age. The patients were selected from the databases of 7 Stroke Units in Belgium. All patients were of central European origin (>90 % were Belgians). Cardiovascular risk factors were recorded. The control group was composed of 326 gender- and ethnicity-matched healthy volunteers without a story of stroke and living in the same area. The study protocol was approved by the ethical committees of all participating hospitals. Written informed consent was obtained from all patients before study entry.

Copenhagen General Population Study and Copenhagen Carotid Stroke Study
This cohort included 750 patients from the greater Copenhagen area referred for carotid ultrasound at Copenhagen University Hospital, during the period 1991 through 2002, for outpatient ultrasonography of the carotid artery. Experienced neurologists and vascular surgeons diagnosed ischemic cerebrovascular disease on the basis of ischemic stroke (n=464) or transient ischemic attack or amarousis fugax (n=266), together with at least 50% stenosis of a carotid artery.[22] Hemorrhage was excluded on computed tomography. The 1545 healthy controls were from the Copenhagen General Population Study.[23]

Edinburgh Stroke Study (ESS)
Consecutive consenting patients with stroke who were admitted to or seen as outpatients at the Western General Hospital, Edinburgh between 2002 and 2005 were prospectively recruited. Cases in this study were those with a clinically evident stroke, demonstrated by brain imaging (CT or MRI) to be ischaemic. An experienced stroke physician assessed each patient as soon as possible after the stroke, prospectively recording demographic and clinical details, including vascular risk factors and results of brain imaging and other investigations. 726 cases were included in the WTCCC2 ischaemic stroke case-control study and contribute to the discovery component of this study, while 276 cases were included as a replication study. Controls for the Edinburgh replication study are 987 stroke-free subjects drawn from The Lothian Birth Cohort 1936,[24] currently living in the Lothian area of Scotland (mainly in Edinburgh).

Glasgow
Patients with ischemic stroke attending the cerebrovascular service of the Western Infirmary, Glasgow, were recruited between 1990 and 2004 as part of an ongoing study of genetic and circulating biomarkers in stroke. All patients underwent brain imaging and extracranial carotid ultrasound in accordance with a standard clinical protocol. The study was approved by the West Ethics Committee.

Genetics of Diabetes Audit and Research in Tayside Study (Go-Darts)
The recruitment of the Diabetes Audit and Research in Tayside Study (DARTS) and the subsequent Genetics of DART study (Go-DARTS) are described in detail elsewhere.[25,26] Briefly, the Genetics of Diabetes Audit and Research in Tayside Study (Go-DARTS) comprises 17,602 participants enrolled between December 1998 and May 2009 in which there are approximately equal numbers of participants with and without a diagnosis of type 2 diabetes mellitus. Relevant clinical data for all Go-DARTS participants are drawn from electronic records of hospital admissions (Scottish Morbidity Register, SMR), deaths (General Registry Office, GRO), biochemical tests and dispensed drug prescriptions, available for the Tayside region. Approval for the current analysis was given by the Go-

DARTS access committee, under standard operating procedures approved by Tayside Research Ethics Committee. Ischaemic stroke events were identified from hospital admissions and death records using the following ICD9 433, 434 and ICD10 I63, I64 and G459. Controls were defined as individuals free of any haemorrhagic and, or ischaemic stroke and, or coronary artery disease and, or lower extremity arterial disease.

Graz Stroke Study
Between 2002 and 2007 white patients with ischemic strokes admitted to the stroke unit of the Medical University Graz Department of Neurology were included. All patients underwent either CT or MRI of the brain and a standardized protocol including a laboratory examination and carotid ultrasound or magnetic resonance angiography and ECG. More extensive cardiac examination, including transesophageal echocardiography or transthoracic echocardiography and Holter, was done in subjects with suspected cardiac embolism. A total of 657 stroke patients were included in the study. The Stroke subtype was assessed according to modified Trial of Org 10172 in Acute Stroke Treatment (TOAST) criteria. Subtyping was done by trained stroke neurologists.

Controls were participants of the Austrian Stroke Prevention Study[27] and came from the same catchment area. All control subjects were recruited between 1991 and 2000 and were free of stroke and dementia. They underwent full risk factor assessment, brain MRI, Duplex scanning of the carotid arteries, ECG, and transthoracic echocardiography.

Interstroke
INTERSTROKE is an international, multicenter case-control study. Cases are patients with a first stroke within 72 h of hospital presentation in whom CT or MRI is performed. Proxy respondents are used for cases unable to communicate. Etiological and topographical stroke subtype is documented for all cases. Controls are hospital- and community-based, matched for gender, ethnicity and age (±5 years). A questionnaire (cases and controls) is used to acquire information on known and proposed risk factors for stroke. Cardiovascular (e.g. blood pressure) and anthropometric (e.g. waist-to-hip ratio) measurements are obtained at the time of interview. Nonfasting blood samples and random urine samples are obtained from cases and controls.

Leuven
Cerebral ischemia, defined as a clinical stroke with imaging confirmation or a TIA with a new ischemic lesion on diffusion weighted MRI, who were admitted to the Stroke Unit of the University Hospitals in Leuven. All patients underwent brain imaging (MRI in 91% of patients, CT in the remainder) and a standardized protocol including carotid ultrasound or CT angiography and cardiac examination (echocardiography and Holter monitoring) in all patients. Control individuals were selected from the same population and were either spouses of patients with multiple sclerosis, amyotrophic lateral sclerosis or stroke or healthy community dwelling subjects partially from the Leuven University Gerontology Database. Controls either confirmed they never had a stroke or TIA or responded negative to any item of the Verification of Stroke Free Status questionnaire.

Lund Stroke Register, Sweden
Lund Stroke Register (LSR) since 2001 continuously enrols patients aged 18 and older with first-ever stroke, living in the primary uptake area of Skåne University Hospital, Lund. The study is mainly hospital-based but has a good coverage of the whole geographical population.[28] All included patients are examined with CT/MR or autopsy of the brain. When clinically indicated, the patients are examined with ultrasound imaging of carotid arteries, echocardiography, and angiography. In this study, first-ever ischemic stroke patients from LSR between 2001 and 2002 were included as well as control subjects from the same geographical uptake area. All patients were assessed by a neurologically trained physician regarding stroke type. The control subjects were individuals without stroke, randomly selected from the official Swedish Population Register and matched for age and gender to the stroke patients. Presence of hypertension, diabetes mellitus, heart disease, smoking and hypercholesterolemia was registered for patients and control subjects. Informed consent was obtained from all individuals or when they were not able to respond from their next-of-kin. The study was approved by the Ethics Committee of Lund University.

Münster (Westphalian Stroke Cases and Controls from the Dortmund Health Study, Germany)
In Westphalia patients were recruited through hospitals participating in the regional Westphalian Stroke Register,[29] located in the west of the country. For this analysis ischemic stroke patients recruited during the period 2000-2005 were included. The register's standardized patient documentation form

included major stroke type and severity, comorbidities, diagnostic and therapeutic details of the treatment process. Ischemic stroke was further subtyped according to the TOAST classification by the documenting physician. Patients who had experienced a transient ischaemic attack or a haemorrhagic stroke were excluded from this analysis.
Controls were drawn from the population based, prospective Dortmund Health Study (DHS),[30] conducted in the same region. Aim of the study was the assessment of the prevalence and incidence of different headache types as well as other chronic health conditions and to analyse their consequences on daily activities of those affected. Participants were randomly drawn from the registration office in the city of Dortmund, within the age range 25 to 74 years and stratified by gender. They participated in a face to face health interview and several physical examinations in the baseline assessment in 2003/4. Cases with a history of stroke (n=28) were excluded from this analysis.
Both studies were approved by the ethics committee of the University of Muenster. All participants gave their informed consent.

Poland:Krakow
Patients were recruited in the stroke unit of the Jagiellonian University in Krakow, Poland (a single-center study). All stroke patients and controls were ≥18 years of age and were white.

All patients had clinically relevant diagnostic workup performed, including brain imaging with computed tomography (CT) (100%) and/or magnetic resonance imaging (MRI) (8%) as well as ancillary diagnostic investigations including duplex ultrasonography of the carotid and vertebral arteries (85.2%), echocardiography (54.8%). MR-angiography, CT-angiography Holter monitoring, transesophageal echocardiography and blood tests for hypercoagulability were performed were indicated. Patients were classified into etiologic subtypes according to the Trial of Org 10172 in Acute Stroke Treatment (TOAST).

The control group included unrelated subjects taken from the population of southern Poland. Control subjects had no apparent neurological disease based on the findings in a structured questionnaire and a neurological examination.

The study was approved by local research ethics committees and informed consent was obtained from all participants.

Portugal
The Portuguese stroke cases and controls used in this study were ascertained and collected as described previously.[31] Briefly, unrelated patients with a clinical diagnosis of IS, who were under the age of 65 at stroke onset, were recruited through Neurology and Internal Medicine Departments throughout Portugal. All patients were seen, and all neuroradiology tests were reviewed by study neurologists. Trauma, tumors, infection, and other causes of neurological deficit were excluded. Since stroke is a late-onset disease, the control group was selected from a group of healthy volunteers with a higher mean age than the case group, thus minimizing the chances for mis-classification as "stroke-free". Control individuals were verified to be free of stroke by direct interview before recruitment, but no brain imaging studies were performed. All participants were adults of Portuguese Caucasian origin. The research protocol was approved by the Ethics Committees of participating institutions, and all participants provided informed consent.

Risk Assessment of Cerebrovascular Events (RACE) Study, Pakistan
RACE is a retrospective case-control study designed to identify and evaluate genetic, lifestyle and biomarker determinants of stroke and its subtype in Pakistan. Samples were recruited from six hospital centres in Pakistan. Cases were eligible for inclusion in the study if they: (1) are aged at least 18 years; (2) presented with a sudden onset of neurological deficit affecting a vascular territory with sustained deficit at 24 hours verified by medical attention within 72 hours after onset (onset is defined by when the patient was last seen normal and not when found with deficit); (3) the diagnosis was supported by CT/MRI; and (4) presented with a Modified Rankin Score of < 2 prior to the stroke. TOAST and Oxfordshire classification systems were used to sub-phenotype all stroke cases. Control participants were individuals enrolled in the Pakistan Risk of Myocardial Infarction Study (PROMIS), a case-control study of acute MI based in Pakistan. Controls in PROMIS were recruited following procedures and inclusion criteria as adopted for RACE cases. In order to minimize any potential selection biases, PROMIS controls selected for this stroke study were frequency matched to RACE cases based on age and gender and were recruited in the following order of priority: (1) non-blood related or blood related

visitors of patients of the out-patient department; (2) non-blood related visitors of stroke patients; (3) patients of the out-patient department presenting with minor complaints.

SMART-study, the Netherlands

Patients aged 18 to 79 years, newly referred to the University Medical Center Utrecht, The Netherlands, with classical risk factors for arterial disease (hypertension, hyperlipidaemia, diabetes mellitus) or with symptomatic arterial disease (coronary heart disease, cerebrovascular disease, abdominal aortic aneurysm, or peripheral arterial obstructive disease) were included in the Second Manifestations of ARTerial disease (SMART) study. A detailed description of the study was published previously.[32] Briefly, patients who gave their written informed consent underwent a standardised vascular screening programme, including a health questionnaire, laboratory assessment, and ultrasonography to investigate the prevalence of additional vascular diseases. The Ethics Committee of the hospital approved the study. For the current study we genotyped a total of 8246 consecutive patients. These patients were included between September 1996 and March 2010 and followed until March 2010 or death.[33] We included all patients as cases who suffered from an ischaemic stroke at baseline of this study or during follow-up (n=623). The remaining patients were used as controls, with exclusion of 911 patients included with transient ischaemic attacks, retinal ischaemia or a stroke in their medical history (n=6712).

South Stockholm Ischemic Stroke Study, Stockholm, Sweden

Swedish patients with ischemic stroke attending the stroke unit or the stroke outpatient clinic at Karolinska University Hospital, Huddinge unit in Stockholm, Sweden, were recruited from 1996 to 2002 as part of an ongoing genetic epidemiology study, the South Stockholm Ischemic Stroke Study (SSISS). The Swedish controls used in this study are population-based controls recruited from the same region in central Sweden as the patients, representing the general population in this area. The individuals were either blood donors recruited at the Huddinge or Karolinska University Hospitals or healthy volunteers (recruited in 1990-1994) recruited by the Clinical Chemistry Department at the Karolinska University Hospital to represent a normal reference. The study was approved by the Bioethics Committee of the Karolinska Institute.

VISP

The VISP trial (P.I. **James Toole, MD**, Wake Forest University School of Medicine (WFU); R01 NS34447) was a multi-center, double-blind, randomized, controlled clinical trial that enrolled patients aged 35 or older with Homocysteine levels above the 25th percentile at screening and a non-disabling cerebral infarction (NDCI) within 120 days of randomization.[34,35] NDCI was defined as an ischemic brain infarction not due to embolism from a cardiac source, characterized by the sudden onset of a neurological deficit. The deficit must have persisted for at least 24 hours, or if not, an infarction in the part of the brain corresponding to the symptoms must have been demonstrated by CT or MRI imaging. The trial was designed to determine if daily intake of a multivitamin tablet with high dose folic acid, vitamin B6 and vitamin B12 reduced recurrent cerebral infarction (1° endpoint), and nonfatal myocardial infarction (MI) or mortality (2° endpoints). Subjects were randomly assigned to receive daily doses of the high-dose formulation (n=1,827), containing 25mg pyridoxine (B6), 0.4mg cobalamin (B12), and 2.5mg folic acid; or the low-dose formulation (n=1,853), containing 200μg pyridoxine, 6μg cobalamin and 20μg folic acid. Enrollment in VISP began in August 1997, and was completed in December 2001, with 3,680 participants enrolled, from 55 clinic sites across the US and Canada and one site in Scotland.

WHI

The WHI Hormone Trial HT (WHI-HT) consisted of two separate clinical trials in postmenopausal women ages 50 to 79 years at baseline—a trial of combined estrogen and progestin (Estrogen plus Progestin or E+P) in women who had an intact uterus at baseline (n=16,608) and a trial of estrogen (Estrogen Alone or E-Alone) in women who had a prior hysterectomy at baseline (n=10,739). Postmenopausal women who gave written informed consent were enrolled in the WHI at 40 clinical centers in the United States. Exclusions for safety reasons included prior diagnosis of breast cancer or other cancers within the past 10 years (except nonmelanoma skin cancer). Women with systolic blood pressure (SBP) of 200 mm Hg or higher or diastolic blood pressure (DBP) of 105 mm Hg or higher were advised to see their physician within a specified period depending on blood pressure level and were temporarily excluded from the clinical trials until their blood pressure was determined to be under control. Stroke diagnosis requiring and/or occurring during hospitalization was based on rapid onset of a neurological deficit attributable to an obstruction or rupture of an arterial vessel system. Hospitalized incident stroke events were identified by semiannual questionnaires and adjudicated following medical

record review, which occurred both locally and centrally. Ischemic strokes were further classified by the central neurologist adjudicators according to the Oxfordshire and Trial of Org 10172 Acute Stroke Trial (TOAST) http://jama.jamanetwork.com.offcampus.lib.washington.edu/article.aspx?articleid=196626 - REF-JOC30333-25 criteria to examine stroke subtypes. The TOAST classification focuses on the presumed underlying stroke mechanism and requires detailed investigations (such as brain computed tomography, magnetic resonance imaging, angiography, carotid ultrasound, and echocardiography).

Genotyping and quality control in Replication studies

European Replication (Barcelona, Belgium (BSS), Glasgow, Graz, Leuven, Lund, Krakow)
Genotyping of European replication samples was carried out at the WTSI using Sequenom iPLEX Gold assays. Individual samples were excluded from analysis if they had call rates of <80% or if reported gender was discordant with gender-specific markers. Pairs of samples showing concordance indicative of being duplicates were referred back to the originating cohort PI for confirmation of individual status, and where duplicates were identified one of each pair was removed.

Genome-wide genotyping of Graz controls was performed at the Human Genotyping Facility, Genetic Laboratory Department of Internal Medicine, Erasmus MC, Rotterdam, The Netherlands. Genotyping platform was the Human610-Quad BeadChip (Illumina). Data were imputed to the 2.5 million autosomal single-nucleotide polymorphisms (SNPs) using HapMap CEU as the reference population. The set of genotyped input SNPs used for imputation was selected based on the highest quality GWA data. We used a callrate >95%, a minor allele frequency >0.01, a Hardy-Weinberg p>1x10-6 and a test of differential missingness by the "mishap" test in PLINK p>1x10-9. We used the Markov Chain Haplotyping (MaCH) package (http://www.sph.umich.edu/csg/abecasis/MACH) version 1.0.15 software and imputed to plus strand of NCBI build 36, HapMap release #22 program. For each imputed SNP a reliability of imputation was estimated (as the ratio of the empirically observed dosage variance to the expected binomial dosage variance: O/E ratio).
Controls for the Glasgow cohort were taken from the Edinburgh Stroke Study, described below.

Copenhagen
Samples were genotyped at Department of Clinical Biochemistry, Herlev Hospital, Copenhagen University Hospital (own lab) using TaqMan assays with call rates at 99% or above. Genotypes were in Hardy-Weinberg equilibrium.

Edinburgh Stroke Study (ESS)
Cases and controls were genotyped at the Wellcome Trust Clinical Research Facility genetics core laboratory at the Western General Hospital, Edinburgh. Cases for the replication study were genotyped on the ABI 7900HT platform, using Applied Biosystems Taqman 6.0. Controls were genotyped for 599 011 common SNPs using the Illumina610-Quadv1 chip (Illumina, Inc., San Diego, CA, USA). Individuals were excluded based on unresolved gender discrepancy, relatedness, call rate (≤0.95) and evidence of non-Caucasian descent. SNPs were included if they met the following conditions: call rate ≥ 0.98, minor allele frequency ≥ 0.01 and Hardy–Weinberg equilibrium test with p ≥ 0.001.[36]

Genetics of Diabetes Audit and Research in Tayside Study (Go-Darts)
Individuals in the Go-DARTS population have been genotyped on both the Affymetrix 6.0 SNP genotyping array and the CardioMetabo Chip, genotyping data were combined across these two platforms to maximise the number of individuals available in each SNP association analysis. 4000 individuals were genotyped on the Affymetrix 6.0 SNP genotyping array and these were imputed to HapMap2 and a British reference panel of 6000 individuals typed on the Illumina 1M dual. An additional 7000 individuals were typed on the Cardio-Metabochip (Illumina). QC was performed in each array population separately: population outliers and one individual per related pair were removed, a minor allele frequency of greater than 0.01 was applied to both arrays, a call rate of greater than 95% was applied to each array, an HWE cut off of 1E-06 was applied to the Affymetrix 6.0 and 1E-04 was applied to the Cardio-Metabochip. Threshold called (0.9) imputed data were combined with directly typed data and the SNPs were tested for association with the stroke phenotypes.

Interstroke
Samples were genotyped at McMaster University using the Illumina Cardiometabochip (Illumina, San Diego, CA, USA). Briefly, samples with a genotype call rate < 97% and SNPs a call rate <90% were excluded. Samples were also excluded due to unexpected duplicates, gender discrepancy, unexpected relatedness or discrepancy between self-reported and genetic ancestry based on principal components analysis. Individual SNPs were excluded from analysis if they had excessive deviation from Hardy-Weinberg Equilibrium proportions ($P < 1.0x10^{-6}$) in any ethnic group or minor allele frequency < 1% in all ethnic groups. Departure from HWE was assessed by an exact method among controls only.

Münster (Westphalian Stroke Cases and Controls from the Dortmund Health Study, Germany)
Samples were genotyped at the Helmholtz Centre, Munich, using the iPLEX Gold Method (Sequenom). Average call rate of the variants measured was >96%. Samples were excluded due to unexpected duplicates (9 samples) and gender discrepancy (60 samples). Individual SNPs were assessed for deviation from Hardy-Weinberg equilibrium (p<1e-5) and minor allele frequency (MAF<0.01). All of the variants tested passed these filters.

Portugal
The Portuguese samples were genotyped in the Genomics Unit of the Instituto Gulbenkian de Ciência using Sequenom's (San Diego, USA) iPlex assay following manufacturer's protocol, and detected in a Sequenom MassArray K2 platform. Extensive quality control was performed using eight HapMap controls of diverse ethnic affiliation, sample duplication within and across plates, non-Mendelian inheritance check in three large pedigrees, Hardy–Weinberg equilibrium (HWE) in the control group (P > 0.05), and a minimum of 90% call rate. Genotype determinations were performed blinded to affection status.

Risk Assessment of Cerebrovascular Events (RACE) Study, Pakistan
All Samples were genotyped on the Illumina Human660W-Quad BeadChip but at two different centres. PROMIS controls were genotyped at the Wellcome Trust Sanger Institute (Sanger) and RACE cases were genotyped at the Center for Inherited Disease Research (CIDR). The raw scan data for cases and controls were clustered together at CIDR to obtain a combined set of genotypes using Illumina GenomeStudio 2010 v2, genotyping module 1.7.4 and Gentrain version 1.0.

SNPs missing with more than 5% of samples were excluded, as were SNPs failing the Hardy-Weinberg equilibrium test at $p < 1.0 \times 10^{-7}$ and SNPs with minor allele frequency less than 1%. All samples with less than 95% call rate were removed from the study. Gender checking was performed using PLINK, and samples with discrepancy between their reported sex and genetic sex were removed from the study. IBD sharing between samples were assessed to identify related samples, and one of each pair of the related samples was excluded from the study.

SMART-study, the Netherlands
Genotyping the 12 snps was performed by KBiosciences (Hoddesdon, United Kingdom). All 12 SNPs had a missingness <3.40% and a Hardy-Weinberg equilibrium p-value > 0.027.

South Stockholm Ischemic Stroke Study, Stockholm, Sweden
Samples were genotyped at deCODE genetics in Reykjavik, Iceland, using the Centaurus (Nanogen) platform (Kutyavin IV, Milesi D, Belousov Y et al. A novel endonuclease IV post-PCR genotyping system. Nucleic Acids Res. 2006;34:e128). The quality of each SNP assay was evaluated by comparing the genotyping of the CEU HapMap samples with the publicly available HapMap data. All SNPs passed mismatch tests, linkage disequilibrium (LD) tests and were in Hardy-Weinberg equilibrium.

VISP
A subset of VISP participants gave consent and were included in the GWAS component of VISP, supported by the National Human Genome Research Institute (NHGRI), Grant U01 HG005160, as part of the Genomics and Randomized Trials Network (GARNET). Samples were genotyped at the Johns Hopkins Center for Inherited Disease Research (CIDR), and genotyping was performed using the Illumina HumanOmni1-Quad_v1-0_B BeadChip (Illumina, San Diego, CA, USA). Individuals were excluded if they were unexpected duplicates or had gender discrepancies.

All VISP participants are stroke cases, therefore we obtained GWAS data (dbGAP) for 1047 external controls from the High Density SNP Association Analysis of Melanoma: Case-Control and Outcomes Investigation (Study Accession: phs000187.v1.p1). These samples were also genotyped on the Illumina HumanOmni1-Quad.

WHI
WHI-GARNET participants were genotyped using the Ilumina Omni-quad chip at the Broad Institute, and imputation was performed into 1000 Genomes at the GARNET Coordinating Center (University of Washington) using BEAGLE. All SNPs passed QC and were in Hardy-Weinberg equilibrium. Association testing for typed or imputed SNPs was performed using PLINK.

References

1 The ARIC investigators. The Atherosclerosis Risk in Communities (ARIC) Study: design and objectives. *Am J Epidemiol* 1989;**129** :687–702.

2 Rosamond WD, Folsom AR, Chambless LE, et al. Stroke incidence and survival among middle-aged adults: 9-year follow-up of the Atherosclerosis Risk in Communities (ARIC) cohort. *Stroke* 1999;**30** :736–743.

3 The National Survey of Stroke. National Institute of Neurological and Communicative Disorders and Stroke. *Stroke*. 1981;**12** :I1–91

4 Ay H, Furie KL, Singhal A, Smith WS, Sorensen AG, Koroshetz WJ. An evidence-based causative classification system for acute ischemic stroke. *Ann Neurol*. 2005;**58** :688–697

5 Yadav S, Schanz R, Maheshwari A, et al. Bio-Repository of DNA in stroke (BRAINS): a study protocol. *BMC Med Genet* 2011; **12**: 34.

6 Fried LP, Borhani NO, Enright P, et al. The Cardiovascular Health Study: design and rationale. *Ann Epidemiol* 1991; **1**: 263–76.

7 Dawber TR, Kannel WB. The Framingham study. An epidemiological approach to coronary heart disease. Circulation 1966;34:553–555.

8 Feinleib M, Kannel WB, Garrison RJ, McNamara PM, Castelli WP. The Framingham Offspring Study. Design and preliminary data. Prev Med 1975;4:518–525.

9 Splansky GL, Corey D, Yang Q et al. The Third Generation Cohort of the National Heart, Lung, and Blood Institute's Framingham Heart Study: design, recruitment, and initial examination. Am J Epidemiol 2007;165:1328–1335.

10 Carandang R, Seshadri S, Beiser A et al. Trends in incidence, lifetime risk, severity, and 30-day mortality of stroke over the past 50 years. *JAMA* 2006;**296**:2939–2946.

11 Seshadri S, Beiser A, Kelly-Hayes M et al. The lifetime risk of stroke: estimates from the Framingham Study. *Strok*e 2006;**37**:345–350.

12 Wolf PA, Kannel WB, Dawber TR. Prospective investigations: the Framingham study and the epidemiology of stroke. *Adv Neurol* 1978;**19**:107–120.

13 Psaty, B.M., et al., The risk of myocardial infarction associated with the combined use of estrogens and progestins in postmenopausal women. *Arch Intern Med* 1994;**154** :1333–9.

14 Psaty, B.M., et al., The risk of myocardial infarction associated with antihypertensive drug therapies. *JAMA*, 1995;**274** :620–5.

15 Klungel, O.H., et al. Antihypertensive drug therapies and the risk of ischemic stroke. Arch *Intern Med*. 2001;161 :37–43.

16 Price TR, Psaty BM, O'Leary D, et al. Assessment of cerebrovascular disease in the Cardiovascular Health Study. *Ann Epidemiol*. 1993;**3**: 504–507.

17 Meschia JF, Nalls M, Matarin M, et al. Siblings with ischemic stroke study: results of a genome-wide scan for stroke loci. *Stroke* 2011; **42**: 2726–32.

18 Chen DT, Worrall BB, Brown RD, Jr, et al. The impact of privacy protections on recruitment in a multicenter stroke genetics study. *Neurology* 2005; **64**: 721–4.

19 Meschia JF, Barrett KM, Chukwudelunzu F, et al. Interobserver agreement in the trial of org 10172 in acute stroke treatment classification of stroke based on retrospective medical record review. *J Stroke Cerebrovasc Dis* 2006; **15**: 266–72.

20 Meschia JF, Brott TG, Brown RD,Jr, et al. The Ischemic Stroke Genetics Study (ISGS) Protocol. *BMC Neurol* 2003; **3**: 4.

21 Hofman A, van Duijn CM, Franco OH, et al. The Rotterdam Study: 2012 objectives and design update. *Eur J Epidemiol* 2011; **26**: 657–86.

22 Zacho J, Tybjærg-Hansen A, Jensen JS, et al. Genetically elevated C-reactive protein and ischemic vascular disease. *N Engl J Med* 2008; **359:** 1897–908.

23 Nordestgaard BG, Palmer TM, Benn M, Zacho J, Tybjærg-Hansen A, Davey Smith G, Timpson NJ. The effect of elevated body mass index on ischemic heart disease risk: Causal estimates from a Mendelian randomisation approach. *Plos Med* 2012; **9**: e1001212

24 Deary IJ, Gow AJ, Taylor MD, et al. The Lothian Birth Cohort 1936: a study to examine influences on cognitive ageing from age 11 to age 70 and beyond. *BMC Geriatrics* 2007; **7**: 28.

25 Doney AS, Dannfald J, Kimber CH, et al. The FTO gene is associated with an atherogenic lipid profile and myocardial infarction in patients with type 2 diabetes: a Genetics of Diabetes Audit and Research Study in Tayside Scotland (Go-DARTS) study. *Circ Cardiovasc Genet* 2009; **2**: 255–9.

26 Morris AD, Boyle DI, MacAlpine R, et al. The diabetes audit and research in Tayside Scotland (DARTS) study: electronic record linkage to create a diabetes register. *DARTS/MEMO Collaboration*. *BMJ* 1997; **315**: 524–8.

27 Schmidt R, Lechner H, Fazekas F, et al. Assessment of cerebrovascular risk profiles in healthy persons: definition of research goals and the Austrian Stroke Prevention Study (ASPS). *Neuroepidemiology.* 1994; **13**: 308–313

28 Hallström B, Jönsson AC, Nerbrand C, Petersen B, Norrving B, Lindgren A. Lund stroke register: Hospitalization pattern and yield of different screening methods for first-ever stroke. Acta Neurol Scand. 2007; **115**: 49–54

29 Berger K, Stögbauer F, Stoll, M, et al. The glu298asp polymorphism in the nitric oxide synthase 3 gene is associated with the risk of ischemic stroke in two large independent case-control studies. *Hum Genet* 2007; **121:** 169–78

30 Vennemann MMT, Hummel T, Berger K: The association between smoking and smell and taste impairment in the general population. *J Neurol* 2008; **255;** 1121–1126

31 Krug T, Manso H, Gouveia L, et al. Kalirin: a novel genetic risk factor for ischemic stroke. *Hum Genet* 2010; **127**: 513–23.

32 Simons PC, Algra A, van de Laak MF, et al. Second manifestations of ARTerial disease (SMART) study: rationale and design. *Eur J Epidemiol*. 1999; **15**: 773–781.

33 Achterberg S, Cramer MJ, Kappelle LJ et al. Patients with coronary, cerebrovascular or peripheral arterial obstructive disease differ in risk for new vascular events and mortality: the SMART study. *Eur J Cardiovasc Prev Rehabil*. 2010; **17**: 424–430.

34 Spence JD, Howard VJ, Chambless LE, et al. Vitamin Intervention for Stroke Prevention (VISP) Trial: Rationale and Design. *Neuroepidemiology*, 2001; **20**:16–25.

35 Toole JF. Vitamin intervention for stroke prevention. *J Neurol Sci* 2002; **203-204**: 121–4.

36 Davies G, Tenesa A, Payton A, et al. Genome-wide association studies establish that human intelligence is highly heritable and polygenic. *Mol. Psychiatry* 2011; **16**: 996–1005.

Acknowledgements

Wellcome Trust Case Control consortium 2

The principal funding for this study was provided by the Wellcome Trust, as part of the Wellcome Trust Case Control Consortium 2 project (085475/B/08/Z and 085475/Z/08/Z).

We also thank S. Bertrand, J. Bryant, S.L. Clark, J.S. Conquer, T. Dibling, J.C. Eldred, S. Gamble, C. Hind, M.L. Perez, C.R. Stribling, S. Taylor and A. Wilk of the Wellcome Trust Sanger Institute's Sample and Genotyping Facilities for technical assistance.

We acknowledge use of the British 1958 Birth Cohort DNA collection, funded by the Medical Research Council grant G0000934 and the Wellcome Trust grant 068545/Z/02, and of the UK National Blood Service controls funded by the Wellcome Trust.

Membership of Wellcome Trust Case Control Consortium 2

Management Committee

Peter Donnelly (Chair)[1,2], Ines Barroso (Deputy Chair)[3], Jenefer M Blackwell[4, 5], Elvira Bramon[6] , Matthew A Brown[7] , Juan P Casas[8] , Aiden Corvin[9], Panos Deloukas[3], Audrey Duncanson[10], Janusz Jankowski[11], Hugh S Markus[12], Christopher G Mathew[13], Colin NA Palmer[14], Robert Plomin[15], Anna Rautanen[1], Stephen J Sawcer[16], Richard C Trembath[13], Ananth C Viswanathan[17], Nicholas W Wood[18]

Data and Analysis Group

Chris C A Spencer[1], Gavin Band[1], Céline Bellenguez[1], Colin Freeman[1], Garrett Hellenthal[1], Eleni Giannoulatou[1], Matti Pirinen[1], Richard Pearson[1], Amy Strange[1], Zhan Su[1], Damjan Vukcevic[1], Peter Donnelly[1,2]

DNA, Genotyping, Data QC and Informatics Group

Cordelia Langford[3], Sarah E Hunt[3], Sarah Edkins[3], Rhian Gwilliam[3], Hannah Blackburn[3], Suzannah J Bumpstead[3], Serge Dronov[3], Matthew Gillman[3], Emma Gray[3], Naomi Hammond[3], Alagurevathi Jayakumar[3], Owen T McCann[3], Jennifer Liddle[3], Simon C Potter[3], Radhi Ravindrarajah[3], Michelle Ricketts[3], Matthew Waller[3], Paul Weston[3], Sara Widaa[3], Pamela Whittaker[3], Ines Barroso[3], Panos Deloukas[3].

Publications Committee

Christopher G Mathew (Chair)[13], Jenefer M Blackwell[4,5], Matthew A Brown[7], Aiden Corvin[9], Chris C A Spencer[1]

1 Wellcome Trust Centre for Human Genetics, University of Oxford, Roosevelt Drive, Oxford OX3 7BN, UK; 2 Dept Statistics, University of Oxford, Oxford OX1 3TG, UK; 3 Wellcome Trust Sanger Institute, Wellcome Trust Genome Campus, Hinxton, Cambridge CB10 1SA, UK; 4 Telethon Institute for Child Health Research, Centre for Child Health Research, University of Western Australia, 100 Roberts Road, Subiaco, Western Australia 6008; 5 Cambridge Institute for Medical Research, University of Cambridge School of Clinical Medicine, Cambridge CB2 0XY, UK; 6 Department of Psychosis Studies, NIHR Biomedical Research Centre for Mental Health at the Institute of Psychiatry, King's College London and The South London and Maudsley NHS Foundation Trust, Denmark Hill, London SE5 8AF, UK; 7 University of Queensland Diamantina Institute, Brisbane, Queensland, Australia; 8 Dept Epidemiology and Population Health, London School of Hygiene and Tropical

Medicine, London WC1E 7HT and Dept Epidemiology and Public Health, University College London WC1E 6BT, UK; 9 Neuropsychiatric Genetics Research Group, Institute of Molecular Medicine, Trinity College Dublin, Dublin 2, Eire; 10 Molecular and Physiological Sciences, The Wellcome Trust, London NW1 2BE; 11 Department of Oncology, Old Road Campus, University of Oxford, Oxford OX3 7DQ, UK , Digestive Diseases Centre, Leicester Royal Infirmary, Leicester LE7 7HH, UK and Centre for Digestive Diseases, Queen Mary University of London, London E1 2AD, UK; 12 Clinical Neurosciences, St George's University of London, London SW17 0RE; 13 King's College London Dept Medical and Molecular Genetics, King's Health Partners, Guy's Hospital, London SE1 9RT, UK; 14 Biomedical Research Centre, Ninewells Hospital and Medical School, Dundee DD1 9SY, UK; 15 King's College London Social, Genetic and Developmental Psychiatry Centre, Institute of Psychiatry, Denmark Hill, London SE5 8AF, UK; 16 University of Cambridge Dept Clinical Neurosciences, Addenbrooke's Hospital, Cambridge CB2 0QQ, UK; 17 NIHR Biomedical Research Centre for Ophthalmology, Moorfields Eye Hospital NHS Foundation Trust and UCL Institute of Ophthalmology, London EC1V 2PD, UK; 18 Dept Molecular Neuroscience, Institute of Neurology, Queen Square, London WC1N 3BG, UK.

The Australian Stroke Genetics Collaborative

Jane M. Maguire, Ph.D.

School of Nursing and Midwifery, University of Newcastle, New South Wales, Australia

John Attia, M.D.

Centre for Clinical Epidemiology and Biostatistics, School of Medicine and Public Health, University of Newcastle, New South Wales, Australia

Rodney J. Scott, Ph.D.

Centre for Bioinformatics, Biomarker Discovery and Information-Based Medicine, Hunter Medical Research Institute, Newcastle, New South Wales, Australia

Lisa F. Lincz, Ph.D.

Hunter Haematology Research Group, Calvary Mater Newcastle Hospital, Newcastle, Australia

Pablo Moscato, Ph.D.

Centre for Bioinformatics, Biomarker Discovery and Information-Based Medicine, Hunter Medical Research Institute, Newcastle, New South Wales, Australia

Simon A. Koblar, M.D.

Stroke Research Program, School of Medicine, University of Adelaide, South Australia, Australia

Jim Jannes, M.D.

Stroke Research Program, School of Medicine, University of Adelaide, South Australia, Australia

Jonathan W. Sturm, M.D.

Department of Neurosciences, Gosford Hospital, Central Coast Area Health, New South Wales, Australia

Graeme J. Hankey, M.D.

Royal Perth Hospital, Perth, Western Australia, Australia

Ross Baker, M.D.

Royal Perth Hospital, Perth, Western Australia, Australia

Mark W. Parsons, M.D.

Centre for Brain and Mental Health Research, University of Newcastle and Hunter Medical Research Institute, New South Wales, Australia

Mark McEvoy, Ph.D.

Centre for Clinical Epidemiology and Biostatistics, School of Medicine and Public Health, University of Newcastle, New South Wales, Australia

Roseanne Peel, M.Sc.

Centre for Clinical Epidemiology and Biostatistics, School of Medicine and Public Health, University of Newcastle, New South Wales, Australia

Wayne Smith, Ph.D.

Hunter Medical Research Institute and University of Newcastle, New South Wales, Australia

Martin D. Lewis, Ph.D.

Stroke Research Program, School of Medicine, University of Adelaide, South Australia, Australia

Tiffany-Jane Evans, B.Sc (Hons)

Centre for Bioinformatics, Biomarker Discovery and Information-Based Medicine, Hunter Medical Research Institute, Newcastle, New South Wales, Australia

Jonathan Golledge, M.D.

Vascular Biology Unit, School of Medicine and Dentistry, James Cook University, Townsville, Queensland, Australia

Erik Biros, Ph.D.

Vascular Biology Unit, School of Medicine and Dentistry, James Cook University, Townsville, Queensland, Australia

National Institutes of Health

Luigi Ferrucci - Longitudinal Studies Section, National Institute on Aging, National Institutes of Health, Baltimore USA

Andrew Singleton - Laboratory of Neurogenetics, National Institute on Aging, National Institutes of Health, Bethesda USA
